# Sensitivity Analysis for Publication Bias in Diagnostic Meta‐Analysis of Sparsity Using the Copas *t*‐Statistic Selection Function

**DOI:** 10.1002/sim.70465

**Published:** 2026-03-18

**Authors:** Taojun Hu, Yi Zhou, Xiao‐Hua Zhou, Satoshi Hattori

**Affiliations:** ^1^ Department of Biomedical Statistics Graduate School of Medicine, The University of Osaka Osaka Japan; ^2^ Department of Biostatistics School of Public Health, Peking University Beijing China; ^3^ Division of Mathematics and Informatics Graduate School of Human Development and Environment, Kobe University Kobe Japan; ^4^ Beijing International Center for Mathematical Research Peking University Beijing China; ^5^ Integrated Frontier Research for Medical Science Division Institute for Open and Transdisciplinary Research Initiatives (OTRI), The University of Osaka Osaka Japan

**Keywords:** diagnostic studies, meta‐analysis, publication bias, sensitivity analysis, sparse data, summary receiver operating characteristic

## Abstract

Publication bias (PB) poses a significant threat to meta‐analysis of diagnostic studies, as studies yielding significant results are more likely to be published in scientific journals, leading to the synthesized diagnostic capacity possibly being overestimated. Sensitivity analysis provides a flexible method to address PB by assuming different proportions of unpublished studies. Most existing methods addressing PB in meta‐analysis of diagnostic studies are based on the bivariate normal model using normal approximations. However, they are unsuitable for meta‐analysis with sparse data, which is common in diagnostic studies with high sensitivities or specificities. Alternatively, the bivariate binomial model relies on the exact within‐study model and has better finite sample properties. To address PB in the bivariate binomial model, we model the selective publication process of diagnostic studies by extending the Copas *t*‐statistic model and propose the likelihood conditional on published and estimation strategies. Our proposal provides an interpretable way to address PB on the summary receiver operating characteristic curve, an essential tool for synthesizing diagnostic accuracy. We show the practicability of the proposed method on several real‐world meta‐analyses of diagnostic studies and evaluate the performance by simulation studies.

## 1 Introduction

Diagnostic studies have long been utilized to assess the effectiveness of medical tests. These tests aim to determine the presence or absence of certain diseases in patients. For diagnostic studies involving continuous biomarkers, the Receiver Operating Characteristic (ROC) curve and the area under the ROC curve (AUC) offer thorough evaluations of diagnostic accuracy at all possible values of cutoff points of biomarkers. To increase the precision of diagnostic accuracy estimated from individual studies, meta‐analysis plays an important role in synthesizing the reported results from multiple studies and providing a comprehensive understanding of the accuracy of the diagnostic test. Suppose we are interested in the meta‐analysis of diagnostic studies. Diagnostics studies may not report the ROC curve and its AUC and often report only a pair of sensitivity and specificity with some cut‐off value. Since the cut‐off values to define the sensitivity and the specificity are often heterogeneous across studies, they are likely to correlate with each other negatively. Thus, summarizing sensitivity and specificity independently would be less appealing. The Summary Receiver Operating Characteristic (SROC) curve is an alternative way to obtain a summary evaluation of a diagnostic test. The SROC curve consolidates sensitivities and specificities from various studies by portraying the relationship between pairs of sensitivity and 1‐specificity over multiple individual studies [1, 2, 3]. The area under the SROC curve (SAUC) serves as a useful metric to evaluate the synthesized accuracy of diagnostic tests. There are two primary methods to estimate the SROC curve/SAUC. The first one is the bivariate normal model [2], which assumes that the logit‐transformed empirical sensitivity and specificity follow a joint normal distribution, relying on the central limit theorem for asymptotic normality, and estimates the parameters through maximizing the likelihood (ML) which is the product of a series of normal distributions. The other is based on the bivariate binomial model. Bayesian inference [3] and the maximum likelihood method [4] can be used for making inferences with the bivariate binomial model.

Since the bivariate binomial model does not rely on the asymptotic normality of the logit‐transformed empirical sensitivity and specificity in constructing the likelihood function, it enhances finite‐sample performance compared to the bivariate normal model. The bivariate binomial model especially addresses challenges presented by meta‐analysis with sparse data, that is, very few or even zero events occur. Suppose the following situation: we are interested in conducting a meta‐analysis of diagnostic studies with the true sensitivity or specificity being high and nearly 1 and with a small size of diseased or non‐diseased subjects. In such cases, the occurrence of the false positive, the non‐diseased subjects being falsely diagnosed as diseased, or the false negative, the diseased subjects being falsely diagnosed as non‐diseased, may fall into small values or even zero, which is called sparse data in meta‐analysis. The sparse data pose great threats to the bivariate normal model [2]. The reason is twofold: first, in the presence of zero frequencies, the logit‐transformed empirical sensitivity and specificity are not defined. To handle this situation with the bivariate normal model, a continuity correction is necessary and may introduce bias in inference [5]. Second, normal approximations for the logit‐transformed empirical sensitivity and specificity with sparse data are unsuitable. Instead, the bivariate binomial model avoids this issue by using the exact within‐study model, rendering itself more reliable in the presence of sparse data. Thus, the bivariate binomial model is more suitable for sparse meta‐analyses of diagnostic studies. We present examples of real‐world meta‐analyses to show the commonness of sparsity in meta‐analysis of diagnostic studies in Section 4. Note that the two models have been thoroughly compared and discussed in previous studies [6, 7, 8, 9]: Chu et al. [6] suggested the bivariate binomial model can easily replace the bivariate normal model. Harbord et al. [7] showed that the bivariate binomial model and bivariate normal model are highly related under specific parameter transformations. However, Chu et al. [10] commented on their relations and showed that the bivariate binomial model would reduce to the bivariate normal model only given a fixed‐effects alpha parameter. Rosenberger et al. [8] have shown the relative superiority of the bivariate binomial model in sparse data.

Besides the data sparsity, publication bias (PB) is another critical issue that influences the reliability of meta‐analyses. Studies with remarkable findings are more likely to be published, leading to an overrepresentation of positive findings. PB has been extensively studied in the standard univariate meta‐analysis of intervention studies. Methods including graphical methods and quantitative methods based on selection models have been proposed to address PB. We put a brief introduction of these related works in Web Appendix A. Among these methods, Copas [11] introduced a sensitivity analysis method to allow selection functions monotonic with the *t*‐statistic, or equivalently its *p*‐value. Studies with significant results, which are strongly related to small *p*‐values, are more likely published. Thus, Copas *t*‐statistics selection model is a natural choice. To the best of our knowledge, the only method incorporating the Copas *t*‐statistics selection model for PB in meta‐analysis of diagnostic studies is based on the bivariate normal model [12]. To make up for the lack of methods for addressing PB in meta‐analysis with sparse data of diagnostic studies, it is necessary to extend the Copas *t*‐statistics selection model to the bivariate binomial model.

In this paper, we propose a sensitivity analysis method to address PB in sparse meta‐analysis of diagnostic studies based on the Copas *t*‐statistics selection model with the bivariate binomial model. We derive the conditional likelihood given published studies based on the bivariate binomial model to make inferences about PB. In the iteration steps of maximizing the likelihood, intensive calculations of integrals are required in the calculations of the marginal selection probability. To address this computational issue, we propose approximation methods for calculating the marginal probability with high accuracy and small computational costs. We show the applicability of our proposal with both real‐world meta‐analyses of diagnostic studies of sparsity and simulation studies. The remainder of this article is structured as follows: Section 2 provides a concise overview of the bivariate binomial model without considering PB. Section 3 introduces the proposed sensitivity analysis method, which applies the Copas *t*‐statistics selection model to the bivariate binomial model; we also elaborate on our inference approaches. In Section 4, we illustrate our proposed sensitivity analysis with real‐world meta‐analyses. In Section 5, we further substantiate the performance of our proposal with simulation studies. Finally, Section 6 summarizes our methods as well as discusses our proposal with the previous related work.

## 2 Bivariate Binomial Model

### 2.1 Notations

Let us consider a meta‐analysis encompassing S published diagnostic studies. The number of subjects in the s‐th (s=1,⋯,S) study is represented as $n^{\left(s\right)}$. Each study evaluates the diagnostic capacity of the common continuous biomarker to determine the presence of the disease based on study‐specific cut‐off points. Without loss of generality, we assume that larger values of the biomarker indicate that the subject is more likely to contract the disease. Consequently, subjects are classified into the tested positive group, which is denoted by $X_{i}^{\left(s\right)}=1$, if the biomarker is larger than the given cut‐off point, or the tested negative group, which is denoted by $X_{i}^{\left(s\right)}=0$, if the biomarker is lower than that. Let the actual disease status of the subject $i\;\left(i=1,\ldots ,n^{\left(s\right)}\right)$ in the s‐th study be denoted by $D_{i}^{\left(s\right)}$. We denote the number of subjects with test outcome $X_{i}^{\left(s\right)}=x$ and true disease status $D_{i}^{\left(s\right)}=d$ as $N_{\mathrm{xd}}^{\left(s\right)}$, and its realization as $n_{\mathrm{xd}}^{\left(s\right)}$, for x=0,1 and d=0,1. Thus, each study is supposed to offer the information of a 2 × 2 contingency table as Table 1. Within the contingency table, the true positive (TP) is $n_{11}^{\left(s\right)}$; the true negative (TN) is $n_{00}^{\left(s\right)}$; the false negative (FN) and false positive (FP) is $n_{01}^{\left(s\right)}$ and $n_{10}^{\left(s\right)}$, respectively. The total numbers of subjects with and without disease are denoted by $n_{1}^{\left(s\right)}$ and $n_{0}^{\left(s\right)}$ respectively.

**TABLE 1:** 2 × 2 contingency table for meta‐analysis of diagnostic studies.

	$D^{\left(s\right)}=0$	$D^{\left(s\right)}=1$	
$X^{\left(s\right)}=0$	$n_{00}^{\left(s\right)}$	$n_{01}^{\left(s\right)}$	
$X^{\left(s\right)}=1$	$n_{10}^{\left(s\right)}$	$n_{11}^{\left(s\right)}$	
	$n_{0}^{\left(s\right)}$	$n_{1}^{\left(s\right)}$	$n^{\left(s\right)}$

### 2.2 Bivariate Binomial Model

Let the study‐specific True Positive Rate (TPR) and False Positive Rate (FPR) be denoted and defined by $\pi_{1}^{\left(s\right)}=P\left(X_{i}^{\left(s\right)}=1\;|\;D_{i}^{\left(s\right)}=1\right)$ and $\pi_{0}^{\left(s\right)}=P\left(X_{i}^{\left(s\right)}=1\;|\;D_{i}^{\left(s\right)}=0\right)$ respectively. The bivariate binomial model [3, 4] is defined by

(1) $$\begin{array}{l} \pi_{1}^{\left(s\right)}=1-G\left\{-\frac{\theta +\theta^{\left(s\right)}+\left(\alpha +\alpha^{\left(s\right)}\right)/2}{\exp\left(\beta /2\right)}\right\}\\ \pi_{0}^{\left(s\right)}=1-G\left\{-\frac{\theta +\theta^{\left(s\right)}-\left(\alpha +\alpha^{\left(s\right)}\right)/2}{\exp\left(-\beta /2\right)}\right\}, \end{array}$$

where G(⋅) represents a known cumulative distribution function (c.d.f). A commonly used choice for G(⋅) is the standard logistic function, defined as $G\left(x\right)=\frac{1}{1+\exp\left(-x\right)}$. Without specific mentions, we use the standard logistic function for G(⋅) in this paper. Model (1) comes from the distributions of the latent biomarker of diseased ($D_{i}^{\left(s\right)}=1$) and non‐diseased subjects ($D_{i}^{\left(s\right)}=0$) following the logistic distribution. The parameter θ denotes the overall location of the biomarker distribution and $\theta^{\left(s\right)}$ describes the heterogeneity from cut‐off values and some other sources over studies. The parameter α denotes the difference between the mean of the biomarker distributions of diseased and non‐diseased subjects and $\alpha^{\left(s\right)}$ represents its heterogeneity over studies. A detailed explanation of parameters and interpretation of the bivariate binomial model was given in Appendix A of Hattori and Zhou [13]. Parameters $\theta^{\left(s\right)}$ and $\alpha^{\left(s\right)}$ are random‐effects, which are assumed to follow the bivariate normal distribution, given by

$$\left(\begin{array}{c} \theta^{\left(s\right)}\\ \alpha^{\left(s\right)} \end{array}\right)∼N\left(\left(\begin{array}{l} 0\\ 0 \end{array}\right),\left(\begin{array}{c} \sigma_{\theta }^{2},0\\ 0,\sigma_{\alpha }^{2} \end{array}\right)\right).$$

The within‐study model assumes TP and FP to follow a binomial distribution, defined as $N_{11}^{\left(s\right)}∼\text{Binomial}\left(n_{1}^{\left(s\right)},\pi_{1}^{\left(s\right)}\right)$ and $N_{10}^{\left(s\right)}∼\text{Binomial}\left(n_{0}^{\left(s\right)},\pi_{0}^{\left(s\right)}\right)$, respectively, conditional on $\theta^{\left(s\right)}$ and $\alpha^{\left(s\right)}$. Thus, the likelihood of the bivariate binomial model without accounting for PB is given by

$$L\left(\Theta \right)=\prod_{s=1}^{S}P\left(N_{11}^{\left(s\right)}=n_{11}^{\left(s\right)},N_{10}^{\left(s\right)}=n_{10}^{\left(s\right)},N_{01}^{\left(s\right)}=n_{01}^{\left(s\right)},N_{00}^{\left(s\right)}=n_{00}^{\left(s\right)}\;|\;n_{1}^{\left(s\right)},n_{0}^{\left(s\right)}\right)$$

with

(2) $$\begin{array}{ll} & P\left(N_{11}^{\left(s\right)}=n_{11}^{\left(s\right)},N_{10}^{\left(s\right)}=n_{10}^{\left(s\right)},N_{01}^{\left(s\right)}=n_{01}^{\left(s\right)},N_{00}^{\left(s\right)}=n_{00}^{\left(s\right)}|\;n_{1}^{\left(s\right)},n_{0}^{\left(s\right)}\right)\\ & \;=\prod_{s=1}^{S}\int_{-\infty }^{\infty }\int_{-\infty }^{\infty }P\left(N_{11}^{\left(s\right)}=n_{11}^{\left(s\right)},N_{10}^{\left(s\right)}=n_{10}^{\left(s\right)}|\theta^{\left(s\right)},\alpha^{\left(s\right)}\right)f_{\theta }\left(\theta^{\left(s\right)}\right)f_{\alpha }\left(\alpha^{\left(s\right)}\right)d\theta^{\left(s\right)}d\alpha^{\left(s\right)}\\ & \;=\prod_{s=1}^{S}\int_{-\infty }^{\infty }\int_{-\infty }^{\infty }\prod_{d=0,1}\left(\begin{array}{c} n_{d}^{\left(s\right)}\\ n_{1d}^{\left(s\right)} \end{array}\right){\left\{\pi_{d}^{\left(s\right)}\right\}}^{n_{1d}^{\left(s\right)}}{\left\{1-\pi_{d}^{\left(s\right)}\right\}}^{n_{d}^{\left(s\right)}-n_{1d}^{\left(s\right)}}f_{\theta }\left(\theta^{\left(s\right)}\right)f_{\alpha }\left(\alpha^{\left(s\right)}\right)d\theta^{\left(s\right)}d\alpha^{\left(s\right)}. \end{array}$$

where $\Theta =\left(\theta ,\alpha ,\beta ,\sigma_{\theta },\sigma_{\alpha }\right)$. By integrating (1) over $\left(\theta^{\left(s\right)},\alpha^{\left(s\right)}\right)$, the overall sensitivity and specificity are derived as,

(3) $$\begin{array}{ll} \text{sensitivity} & =\pi_{1}=1-G\left\{-\frac{\theta +\alpha /2}{\exp\left(\beta /2\right)}\right\}\\ \text{specificity} & =1-\pi_{0}=G\left\{-\frac{\theta -\alpha /2}{\exp\left(-\beta /2\right)}\right\}. \end{array}$$

The pair of the overall sensitivity and specificity is also called the summary operating point (SOP) [12]. If we let x represent 1‐specificity, then the sensitivity can be formulated as a function of x with respect to the parameters α and β, after eliminating θ. Thus, the SROC curve, which illustrates the relationship between sensitivity and 1‐specificity, is defined by [13]:

(4) $$\text{SROC}\left(x;\alpha ,\beta \right)=1-G\left\{-\alpha \exp\left(-\beta /2\right)+\exp\left(-\beta \right)G^{-1}\left(1-x\right)\right\},$$

and the SAUC is defined by:

(5) $$\text{SAUC}\left(\alpha ,\beta \right)=\int_{0}^{1}\;\text{SROC}\left(x;\alpha ,\beta \right)\mathrm{dx}.$$

Most of the literature refers to the quantities defined by (4) and (5) as HSROC and HSAUC, respectively. In addition, Reitsma et al. [2] proposed another way to describe the relationship between sensitivity and 1‐specificity, named SROC and its SAUC. In Appendix A of Zhou et al. [12], the authors showed the equivalence between these two quantities. Thus, we refer to these quantities in (4) and (5) as SROC and SAUC instead, to avoid the unnecessary proliferation of terminology.

## 3 Sensitivity Analysis for Publication Bias

### 3.1 Selection Function

In this section, we aim to address PB with the Copas *t*‐statistics selection model for the bivariate binomial model. The bivariate nature of diagnostic studies makes it difficult to directly apply the original Copas t‐statistic selection model. In practice, the funnel plot on the lnDOR, which is defined by logit(spe) + logit(sen), is widely used to detect PB [14, 15]. Thus, the significance (or the t‐statistic) of the lnDOR is naturally selected as the key statistic of the selection model. To allow flexibility, we adopt the idea from Zhou et al. [12] that considers the linear combination of logit‐transformed sensitivity (denoted by logit(sen)) and specificity (denoted by logit(spe)) as the key statistic in the selection model, that is

(6) $$c_{0}\text{logit}\left(\mathrm{spe}\right)+c_{1}\text{logit}\left(\mathrm{sen}\right),$$

where logit(x) is defined as $\log\frac{x}{1-x}$ and $c_{0},c_{1}$ are non‐negative coefficients indicating the weight of sensitivity or specificity in the selective mechanism. Zhou et al. [12] pointed out that the *t*‐statistics are scale‐invariant and they constrained $c_{1}^{2}+c_{0}^{2}=1\;\left(c_{0},c_{1}\in \left[0,1\right]\right)$ without loss of generality (see Web Appendix B). We followed their settings. For the s‐th study, the empirical key statistic is given by $c_{0}\log\left(n_{00}^{\left(s\right)}/n_{01}^{\left(s\right)}\right)+c_{1}\log\left(n_{11}^{\left(s\right)}/n_{10}^{\left(s\right)}\right)$. The empirical variance for (6) is then derived as

$$c_{0}^{2}\left(\frac{1}{n_{01}^{\left(s\right)}}+\frac{1}{n_{00}^{\left(s\right)}}\right)+c_{1}^{2}\left(\frac{1}{n_{11}^{\left(s\right)}}+\frac{1}{n_{10}^{\left(s\right)}}\right)$$

for the s‐th study. Thus, the t‐statistic for (6) is given by

(7) $$t^{\left(s\right)}=\frac{c_{0}\log\frac{n_{00}^{\left(s\right)}}{n_{01}^{\left(s\right)}}+c_{1}\log\frac{n_{11}^{\left(s\right)}}{n_{10}^{\left(s\right)}}}{\sqrt{c_{0}^{2}\left(\frac{1}{n_{01}^{\left(s\right)}}+\frac{1}{n_{00}^{\left(s\right)}}\right)+c_{1}^{2}\left(\frac{1}{n_{11}^{\left(s\right)}}+\frac{1}{n_{10}^{\left(s\right)}}\right)}}.$$

The *t*‐statistics for the lnDOR, sensitivity, specificity correspond to $\left(c_{0},c_{1}\right)=\left(1/\sqrt{2},1/\sqrt{2}\right)$, $\left(c_{0},c_{1}\right)=\left(1,0\right)$, and $\left(c_{0},c_{1}\right)=\left(0,1\right)$, respectively. The selective publication process suggested by the significance of (6) from each study is modeled by the following selection function:

(8) $$P\left(\text{select}\;|\;\gamma_{0},\gamma_{1},t^{\left(s\right)}\right)=a\left(t^{\left(s\right)}\right)=H\left(\gamma_{0}+\gamma_{1}t^{\left(s\right)}\right),$$

where H(⋅) is an arbitrary non‐decreasing function valued from 0 to 1. The probit function Φ(⋅) is adopted in this paper since it often makes the formula derivation simpler and is widely used in previous studies [11, 12]. Equation (8) indicates that when $\left(c_{0},c_{1}\right)=\left(1/\sqrt{2},1/\sqrt{2}\right)$, the selective publication is determined by the significance of the lnDOR; when $\left(c_{0},c_{1}\right)=\left(0,1\right)$ or (1,0), the selective publication is determined by the significance of sensitivity or specificity, respectively. For studies with zero cells in the contingency table, $t^{\left(s\right)}$ is undefined. We apply the continuity correction, which is adding 0.5 to all the cells, and then calculate $t^{\left(s\right)}$. We note that the continuity correction is only used for calculating the study‐specific t‐statistic in the selection function (8) to model the selective publication and is **not** used in the exact likelihood of meta‐analysis.

### 3.2 Exact Conditional Likelihood Function and Strategies of Statistical Inference

In our likelihood‐based sensitivity analysis, we make inferences based on maximizing the likelihood conditional on the published. Previous likelihood‐based sensitivity analysis methods [12, 16, 17] are based on the bivariate normal model, and thus deriving and inferring the conditional likelihood is rather simple based on the nature of the bivariate normal distribution. Our method is based on the bivariate binomial model, where the within‐study model is the exact binomial distribution instead of the normal model. Thus, the likelihood combines both the probability mass function for discrete variables and density functions for continuous variables and involves complex summations of integrals. We need to tackle the computational issues in the estimation.

Based on the bivariate binomial model, the likelihood conditional on the published is derived as

(9) $$\begin{array}{cc} L_{O}\left(\Theta ,\gamma_{0},\gamma_{1}\right) & =\prod_{s=1}^{S}P\left(N_{11}^{\left(s\right)}=n_{11}^{\left(s\right)},N_{01}^{\left(s\right)}=n_{01}^{\left(s\right)},N_{10}^{\left(s\right)}=n_{10}^{\left(s\right)},N_{00}^{\left(s\right)}=n_{00}^{\left(s\right)}\;|\;\text{select}\right)\\ & =\prod_{s=1}^{S}\frac{P\left(\text{select}\;|\;n_{11}^{\left(s\right)},n_{10}^{\left(s\right)},n_{01}^{\left(s\right)},n_{00}^{\left(s\right)}\right)f_{P}\left(n_{11}^{\left(s\right)},n_{10}^{\left(s\right)},n_{01}^{\left(s\right)},n_{00}^{\left(s\right)}\right)f_{P}\left(n_{1}^{\left(s\right)},n_{0}^{\left(s\right)}\right)}{P\left(\text{select}\right)}\\ & =\prod_{s=1}^{S}\frac{P\left(\text{select}\;|\;\gamma_{0},\gamma_{1},t^{\left(s\right)}\right)f_{P}\left(n_{11}^{\left(s\right)},n_{10}^{\left(s\right)},n_{01}^{\left(s\right)},n_{00}^{\left(s\right)}\right)f_{P}\left(n_{1}^{\left(s\right)},n_{0}^{\left(s\right)}\right)}{P\left(\text{select}\right)}\\ & =\prod_{s=1}^{S}\frac{a\left(t^{\left(s\right)}\right)f_{P}\left(n_{11}^{\left(s\right)},n_{10}^{\left(s\right)},n_{01}^{\left(s\right)},n_{00}^{\left(s\right)}\right)f_{P}\left(n_{1}^{\left(s\right)},n_{0}^{\left(s\right)}\right)}{P\left(\text{select}\right)}, \end{array}$$

where $f_{P}\left(n_{11}^{\left(s\right)},n_{10}^{\left(s\right)},n_{01}^{\left(s\right)},n_{00}^{\left(s\right)}\right)$ refers to $P(N_{11}^{\left(s\right)}=n_{11}^{\left(s\right)},N_{10}^{\left(s\right)}=n_{10}^{\left(s\right)},N_{01}^{\left(s\right)}=n_{01}^{\left(s\right)},N_{00}^{\left(s\right)}=n_{00}^{\left(s\right)}|n_{1}^{\left(s\right)},n_{0}^{\left(s\right)})$ in (2), while $f_{P}\left(n_{1}^{\left(s\right)},n_{0}^{\left(s\right)}\right)$ denotes the marginal probability mass function of $\left(n_{1}^{\left(s\right)},n_{0}^{\left(s\right)}\right)$ in the population studies, and the denominator P(select) denotes the marginal selection probability.

We regard the marginal selection probability P(select)=p as the sensitivity parameter in our method. Given a fixed value of p, the probability of $\left(n_{1}^{\left(s\right)},n_{0}^{\left(s\right)}\right)$ conditional on published is derived as

(10) $$f_{O}\left(n_{1}^{\left(s\right)},n_{0}^{\left(s\right)}\right)=P\left(n_{1}^{\left(s\right)},n_{0}^{\left(s\right)}\;|\;\text{select}\right)=\frac{P\left(\text{select}\;|\;n_{1}^{\left(s\right)},n_{0}^{\left(s\right)}\right)f_{P}\left(n_{1}^{\left(s\right)},n_{0}^{\left(s\right)}\right)}{p}.$$

An equivalent form is

$$f_{P}\left(n_{1}^{\left(s\right)},n_{0}^{\left(s\right)}\right)=p\frac{1}{P\left(\text{select}\;|\;n_{1}^{\left(s\right)},n_{0}^{\left(s\right)}\right)}f_{O}\left(n_{1}^{\left(s\right)},n_{0}^{\left(s\right)}\right).$$

Then, integrating it over $\left(n_{1}^{\left(s\right)},n_{0}^{\left(s\right)}\right)$ in both sides, we can derive

(11) $$\frac{1}{p}=E_{O}\left(\frac{1}{P\left(\text{select}\;|\;n_{1}^{\left(s\right)},n_{0}^{\left(s\right)}\right)}\right),$$

where $E_{O}$ means the expectation conditional on the published. We consider the empirical version of (11) by replacing the expectation conditional on published with the average over observed studies, that is

(12) $$\frac{1}{p}=\frac{1}{S}\sum_{s=1}^{S}\frac{1}{P\left(\text{select}\;|\;n_{1}^{\left(s\right)},n_{0}^{\left(s\right)}\right)}.$$

Combining with (10), the likelihood conditional on published (9) can be rewritten as




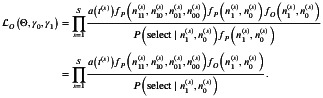




Thus, we can derive the log‐likelihood conditional on the published, that is,

(13) $$\begin{array}{ll} \ell_{O}\left(\Theta ,\gamma_{0},\gamma_{1}\right) & =\sum_{s=1}^{S}\log f_{P}\left(n_{11}^{\left(s\right)},n_{10}^{\left(s\right)},n_{01}^{\left(s\right)},n_{00}^{\left(s\right)}\right)+\sum_{s=1}^{S}\log a\left(t^{\left(s\right)}\right)-\\ & \;\sum_{s=1}^{S}\log P\left(\text{select}|n_{1}^{\left(s\right)},n_{0}^{\left(s\right)}\right)+\sum_{s=1}^{S}\log f_{O}\left(n_{1}^{\left(s\right)},n_{0}^{\left(s\right)}\right). \end{array}$$

Equation (12) poses a constraint on the likelihood. Parameters $\left(\Theta ,\gamma_{0},\gamma_{1}\right)$ can be estimated by maximizing the log‐likelihood (13) with the constraint function (12). The last term of (13) is a constant with respect to the parameters. The first and third terms in the right of the equal sign of (13) are given by (2) and (8), respectively. The term $P\left(\text{select}\;|\;n_{1}^{\left(s\right)},n_{0}^{\left(s\right)}\right)$ is special to the case of the bivariate binomial model and the corresponding part is of much simpler form in the normal‐approximation‐based models. The conventional normal‐approximation‐based models construct this term conditional on the within‐study standard errors for all studies and assume them to be randomly sampled from an unknown distribution. As the bivariate binomial distribution does not involve the within‐study standard errors in the likelihood, we construct this term conditional on the sample sizes, $\left(n_{1}^{\left(s\right)},n_{0}^{\left(s\right)}\right)$, and assume that the sample sizes of published studies are random samples from an unknown distribution.

One difficulty of optimizing (13) lies in the calculation of $P\left(\text{select}\;|\;n_{1}^{\left(s\right)},n_{0}^{\left(s\right)}\right)$ in (13) and (12). Specifically, we can express it by

(14) $$\begin{array}{cc} & P\left(\text{select}\;|\;n_{1}^{\left(s\right)},n_{0}^{\left(s\right)}\right)\\ & \;=\sum_{m_{11}=0}^{n_{1}^{\left(s\right)}}\sum_{m_{00}=0}^{n_{0}^{\left(s\right)}}P\left(\text{select}\;|\;m_{11},n_{1}^{\left(s\right)},m_{00},n_{0}^{\left(s\right)}\right)f_{P}\left(m_{11},n_{1}^{\left(s\right)}-m_{11},n_{0}^{\left(s\right)}-n_{01}^{\left(s\right)},m_{00}\right)\\ & \;=\sum_{m_{11}=0}^{n_{1}^{\left(s\right)}}\sum_{m_{00}=0}^{n_{0}^{\left(s\right)}}a\left(t\left(m_{11},n_{1}^{\left(s\right)},m_{00},n_{0}^{\left(s\right)}\right)\right)f_{P}\left(m_{11},n_{1}^{\left(s\right)}-m_{11},n_{0}^{\left(s\right)}-n_{01}^{\left(s\right)},m_{00}\right), \end{array}$$

with

(15) $$t\left(m_{11},n_{1}^{\left(s\right)},m_{00},n_{0}^{\left(s\right)}\right)=\frac{c_{0}\log\frac{m_{00}}{n_{0}^{\left(s\right)}-m_{00}}+c_{1}\log\frac{m_{11}}{n_{1}^{\left(s\right)}-m_{11}}}{\sqrt{c_{0}^{2}\left(\frac{1}{n_{0}^{\left(s\right)}-m_{00}}+\frac{1}{m_{00}}\right)+c_{1}^{2}\left(\frac{1}{m_{11}}+\frac{1}{n_{1}^{\left(s\right)}-m_{11}}\right)}}.$$

It involves both the selection functions a(⋅) and the probability mass function for the bivariate binomial model $f_{P}\left(\cdot \right)$. Thus, it is a function of $\left(\Theta ,\gamma_{0},\gamma_{1}\right)$. For simplification, we denote $t\left(m_{11},n_{1}^{\left(s\right)},m_{00},n_{0}^{\left(s\right)}\right)$ as ${\tilde{t}}^{\left(s\right)}\left(m_{11},m_{00}\right)$ in the following. As given in (2), $f_{P}\left(\cdot \right)$ involves bivariate integration. Thus, we need to calculate many integrals in (14) and sum them up. This calculation is computationally demanding and may also suffer from the issue of error accumulation. We address the problem through an approximation approach. We regard $\left(m_{11},m_{00}\right)$ as random variables following binomial distributions, that is, $m_{11}∼\text{Binomial}\left(n_{1}^{\left(s\right)},p_{1}^{\left(s\right)}\right)$ and $m_{00}∼\text{Binomial}\left(n_{0}^{\left(s\right)},p_{0}^{\left(s\right)}\right)$. Then (14) can be re‐expressed by $P\left(\text{select}\;|\;n_{1}^{\left(s\right)},n_{0}^{\left(s\right)}\right)=E_{{\tilde{t}}^{\left(s\right)}\left(m_{11},m_{00}\right)}H\left(\gamma_{0}+\gamma_{1}{\tilde{t}}^{\left(s\right)}\left(m_{11},m_{00}\right)\right)$, where $E_{{\tilde{t}}^{\left(s\right)}\left(m_{11},m_{00}\right)}$ implies the expectation with respect to the distribution of ${\tilde{t}}^{\left(s\right)}\left(m_{11},m_{00}\right)$. We use the asymptotic normality properties of the statistics ${\tilde{t}}^{\left(s\right)}\left(m_{11},m_{00}\right)$ to approximate its true distribution and then derive an approximation for $P\left(\text{select}\;|\;n_{1}^{\left(s\right)},n_{0}^{\left(s\right)}\right)$. While the direct calculation for (14) needs continuity correction to compute ${\tilde{t}}^{\left(s\right)}\left(m_{11},m_{00}\right)$ if any of $m_{11},n_{1}^{\left(s\right)}-m_{11},m_{00}$ or $n_{0}^{\left(s\right)}-m_{00}$ is zero, our approximation method avoids using the continuity correction. The proof of the asymptomatic normality for ${\tilde{t}}^{\left(s\right)}\left(m_{11},m_{00}\right)$ and the derivation of its asymptomatic expectation and variance are presented in the Web Appendix C.

To make inferences with the constraint (12), we derive the parameter $\gamma_{0}$ as a function of the remaining parameters based on the constraint function (12) given the fixed value of p, denoted by ${\hat{\gamma }}_{0}={\hat{\gamma }}_{0}\left(\Theta ,\gamma_{1};p\right)$. This is because $P\left(\text{select}\;|\;n_{1}^{\left(s\right)},n_{0}^{\left(s\right)}\right)$ is a function of $\left(\Theta ,\gamma_{0},\gamma_{1}\right)$ and it is easy to show that $P\left(\text{select}\;|\;n_{1}^{\left(s\right)},n_{0}^{\left(s\right)}\right)$ is a monotone function of $\gamma_{0}$ given $\left(\Theta ,\gamma_{1}\right)$ based on the monotonicity of a(⋅), ranging from 0 to 1. Thus, $\gamma_{0}$ can be expressed as a function of $\left(\Theta ,\gamma_{1}\right)$ by solving (12) when p∈(0,1). After eliminating $\gamma_{0}$, the parameters in (13) are left to $\left(\Theta ,\gamma_{1}\right)$. The maximum likelihood estimates (MLE) of the parameters are denoted by $\hat{\Theta },{\hat{\gamma }}_{1}$. We estimate the asymptomatic variance matrix for the parameters Θ through the inverse of the observed Fisher information matrix, denoted by $\hat{\sum }$. The SROC curve and SAUC in the presence of PB can be estimated through the plug‐in of $\hat{\Theta }$ to the expressions of SROC curve (4) and SAUC (5). The resulting estimator for SAUC is denoted by $\hat{\text{SAUC}}=\text{SAUC}\left(\hat{\alpha },\hat{\beta }\right)$. The variance of the SAUC can be estimated through the Delta method, that is 


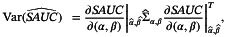


where the superscript T means the transpose of the vector and the ${\hat{\sum }}_{\alpha ,\beta }$ is the submatrix of $\hat{\sum }$ corresponding to the components for (α,β).

We construct a 95% confidence interval (CI), which is always within [0,1] by applying the Delta method with some transformation g from (0,1) to (−∞,∞). The asymptomatic expectation and variance of g‐transformed SAUC are given by $g\left(\hat{\text{SAUC}}\right)$ and ${\left\{g^{\prime }\left(\hat{\text{SAUC}}\right)\right\}}^{2}\mathrm{Var}\left(\hat{\text{SAUC}}\right)$, respectively, where $g^{\prime }=dg\left(x\right)/dx$. The reconstructed 95% CI for SAUC can be expressed as

$$g^{-1}\left\{g\left(\hat{\text{SAUC}}\right)\pm 1.96g^{\prime }\left(\hat{\text{SAUC}}\right)\sqrt{\mathrm{Var}\left(\hat{\text{SAUC}}\right)}\right\}.$$

A common choice for g is the logit function, g(x)=log(x/(1−x)). By varying the values of marginal selection probabilities p within 0 and 1, one could know the impact of PB through the changes of estimates for the parameters and the SAUC.

## 4 Application

We illustrated our sensitivity analysis method with the meta‐analysis conducted by Li et al. [18]. This meta‐analysis evaluated the effectiveness of the neutrophil CD64 expression as a biomarker to differentiate infected patients from non‐infected ones with bacterial infection with 27 individual studies. The data are presented in Table 2. Of the 27 independent studies, two studies (No. 7 and 20) had zero frequencies of FP, and most of the other studies had very low frequencies of FP or FN. In the original paper, Li et al. [18] estimated the SAUC as 0.925 (95% CI: [0.880, 0.954]), highlighting the potential of neutrophil CD64 expression as a promising biomarker for diagnosing bacterial infection. The overall sensitivity and specificity were estimated as 0.819 (95% CI: [0.687, 0.951]) and 0.895 (95% CI: [0.823, 0.967]), respectively. It pointed out that the meta‐analysis suffered much from PB since Egger's test showed a remarkable trend of PB (p<0.001). The original paper did not address how PB could affect the estimate of the SAUC. Zhou et al. [12] used this dataset for their illustration, and the issue of sparsity was overlooked in their analysis. We re‐evaluate the impact of PB in this meta‐analysis using the proposed method. We conducted sensitivity analysis setting p=0.2,0.4,0.6,0.8,1 with three specified selective mechanisms: $\left(c_{0},c_{1}\right)=\left(1/\sqrt{2},1/\sqrt{2}\right),\left(0,1\right)$ and (1,0). Note that p=1 implies the analysis assumes no selective publication, and the estimates should be the same in the bivariate binomial model and our proposal.

**TABLE 2:** Data of meta‐analysis of CD64 study.

Study	Author	TP	FP	FN	TN	Cut‐off points
1	Icardi	53	6	3	47	1.19
2	Cid	100	10	15	7	1.5
3	Gamez‐Diaz	266	73	138	133	1.7
4	Groselj‐Gren	13	6	4	23	1.86
5	Gros	91	16	54	132	2.2
6	Bhandari	89	63	39	102	2.3
7	Groselj‐Gren	17	0	7	12	2.38
8	Groselj‐Gren	19	3	10	24	2.45
9	Dilli	31	6	4	36	4.39
10	Genel	40	8	9	27	3.05 MFI
11	Tang	50	10	14	32	8.5 MFI
12	Hussein	17	2	1	16	43.5 MFI
13	Mokuda	14	2	1	23	1800 mol
14	Nishino	19	2	6	34	2000 mol
15	Doi	19	1	12	67	2000 mol
16	Tanaka	28	2	18	93	2000 mol
17	Matsui	51	7	4	195	2000 mol
18	Allen	23	4	4	40	2000 mol
19	Cardelli	50	3	2	57	2398 mol
20	Livaditi	35	0	2	10	2566 mol
21	Ng	30	7	2	51	4000 mol
22	Hsu	49	1	6	10	4300 mol
23	Lam	107	37	29	137	6010 mol
24	Ng	72	20	21	175	6136 mol
25	Ng	91	25	24	198	6136 mol
26	Tillinger	21	2	1	74	10 000 mol
27	Layseca‐Esp	8	1	23	16	

**FIGURE 1:**
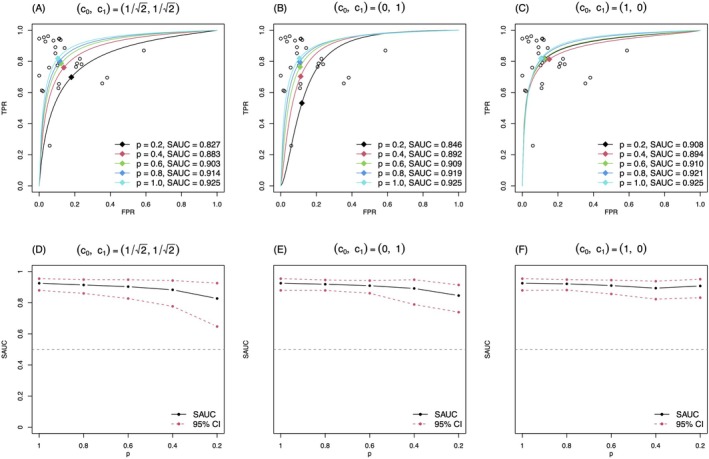
The estimated SROC curves and SAUC under three selective publication mechanisms in CD64 example with the bivariate binomial model (our proposal).

The results of the proposed method are presented in Figure 1. In the upper panels of Figure 1, we depicted the estimated SROC curves under various *p*s with the three settings of $\left(c_{0},c_{1}\right)$. Zhou et al. [12] presented that tracing the SOP, that is the pair of (sensitivity, specificity), over p was useful to characterize what kind of selective process was considered with the supposed selection function. Thus, we also present the SOPs for this purpose. As shown in panel (A) of Figure 1, the plots of the SOPs suggested a selective publication process under which studies around the lower right part of the SROC curve were less likely published. Recall that the SOP of the original analysis (p=1) was (0.819,0.895) and it moved up to (0.755,0.869) with p=0.2. The change of the SROC curve and SAUC suggested that the test accuracy was robust against the selective publication mechanism determined by the significance of the lnDOR. Tracing the SOPs in panels (B) to (C) of Figure 1, one could understand that the selection function with $\left(c_{0},c_{1}\right)=\left(0,1\right)$ and (1,0) modeled different publication mechanisms and the figures indicated that impacts by the selective publication mechanisms determined by only sensitivity or specificity would be minor: SOP was moved to (0.529,0.884) with $\left(c_{0},c_{1}\right)=\left(0,1\right)$ under p=0.2, where specificity only changed by −0.011 from 0.895 to 0.884; and SOP was moved to (0.818,0.891) with $\left(c_{0},c_{1}\right)=\left(1,0\right)$ under p=0.2, where sensitivity only changed by −0.001 from 0.819 to 0.818.

We showed the estimated SAUC with varying p in panels (D) to (F) of Figure 1. The SAUC was 0.925 (95% CI: [0.880, 0.954]) without accounting for selective publication (p=1). With all specified values of p, the lower bounds for SAUCs were larger than 0.5 under all three selective mechanisms, suggesting the effectiveness of neutrophil CD64 expression in diagnosing bacterial infection since SAUC = 0.5 indicated that the diagnostic test result was a random guess. The estimated SAUC under p=0.2 is much lower than that under p=1 under all three selective publication mechanisms, suggesting the potential impact of selective publication for this example; ignoring PB will lead to an overestimation of the SAUC.

**FIGURE 2:**
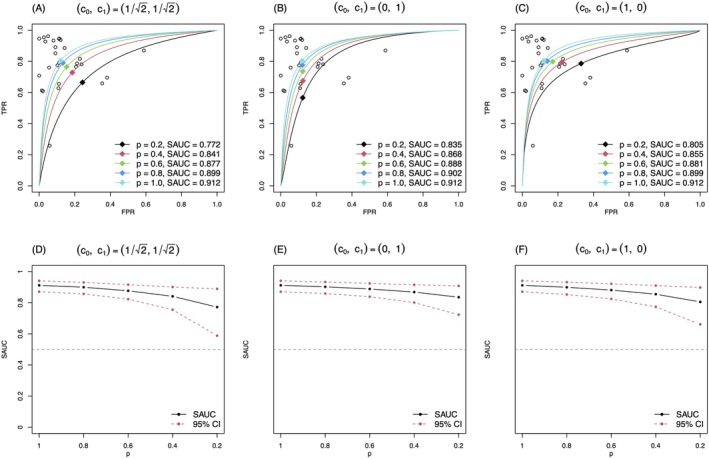
The estimated SROC curves and SAUC under three selective publication mechanisms in CD64 example with the bivariate normal model by Zhou et al. [12].

We also conducted the sensitivity analysis based on the bivariate normal model by Zhou et al. [12] for comparisons. Although the parameterizations between the bivariate binomial and bivariate normal models are different, Harbord et al. [7] showed there was a correspondence between the parameters of the bivariate normal model and those of the bivariate binomial model. Each model has its definition of the SROC curve and SAUC; the bivariate binomial model naturally leads to the SROC curve (4) and SAUC (5), whereas Reitsma et al. [2] introduced an alternative definition of the SROC with the bivariate normal model. With the correspondence between the parameters of the two models, one may derive the SROC curve/SAUC with (4) and (5) even if the bivariate normal model is used for parameter estimation. The formula to this end is given in Equation (A1) of Zhou et al. [12]. The estimated SROC curves with the bivariate normal model were given in panels (A) to (C) of Figure 2, assuming different selective mechanisms under p=0.2,0.4,0.6,0.8,1; the variation of SAUC was shown in panels (D) to (F) of Figure 2. We could observe that the bivariate normal model obtained lower estimates for the SAUC compared with the bivariate binomial model. The differences between Figures 1 and 2 can be attributed to the ignorance of tackling the sparsity by the bivariate normal model. As shown in Figures 1 and 2, using the exact within‐study model instead of applying normal approximation would give a higher estimate for SAUC in this example. For example, the SAUC by the bivariate binomial model with p=0.2 was 0.827 whereas that by the bivariate normal model was 0.771.

We also analyzed two additional meta‐analysis datasets of diagnostic studies to show the wide applicability of our proposal. The results were placed in the Web Appendices D and E.

## 5 Simulation Studies

We evaluated the performance of the proposed methods with simulation studies. We considered three sets of overall sensitivity and specificity in (3): (0.9, 0.5), (0.5, 0.9), and (0.8, 0.8) given the link function G(⋅) as the standard logistic function. With the scale parameter β=0.15 set, we determined three settings of θ and α by solving (3). We set the standard deviation of cut‐off parameters and accuracy parameters $\left(\sigma_{\theta },\sigma_{\alpha }\right)=\left(0.6,1.2\right),\left(1.2,0.6\right)$ to see if the performance of our proposal would be affected by varying the variance parameters. Thus, we considered 6 scenarios for data generation and the parameters are summarized in the Web Appendix F. We considered simulating small‐scale, medium‐scale, and relatively large‐scale, and large‐scale meta‐analyses with $\tilde{S}\;\left(\tilde{S}=15,25,50,100\right)$ published and unpublished studies.

Under each scenario, we generated 1000 independent meta‐analyses with $\tilde{S}$ studies. For each dataset, the number of subjects with the disease, $n_{1}^{\left(s\right)}$, was sampled from a discrete uniform distribution U(10,30), and the number of subjects without the disease, $n_{0}^{\left(s\right)}$, sampled from the U(200,300), mirroring real‐world disparities in subject numbers. The variations in cut‐off and accuracy parameters across studies, $\theta^{\left(s\right)}$ and $\alpha^{\left(s\right)}$, were sampled from normal distributions $N\left(0,\sigma_{\theta }^{2}\right)$ and $N\left(0,\sigma_{\alpha }^{2}\right)$, respectively. Then we set $\pi_{1}^{\left(s\right)}$ and $\pi_{0}^{\left(s\right)}$ according to (1). $n_{11}^{\left(s\right)}$ and $n_{10}^{\left(s\right)}$ were generated from binomial distributions $\text{Binomial}\left(n_{1}^{\left(s\right)},\pi_{1}^{\left(s\right)}\right)$ and $\text{Binomial}\left(n_{0}^{\left(s\right)},\pi_{0}^{\left(s\right)}\right)$, respectively. The sampled $\left(n_{11}^{\left(s\right)}\right),\left(n_{10}^{\left(s\right)}\right)$ and $\left(n_{01}^{\left(s\right)},n_{00}^{\left(s\right)}\right)=\left(n_{1}^{\left(s\right)}-n_{11}^{\left(s\right)},n_{0}^{\left(s\right)}-n_{10}^{\left(s\right)}\right)$ constituted the 2 × 2 contingency tables for both published and unpublished studies.

We considered the selection function based on the *t*‐statistics for the lnDOR, which corresponded to $\left(c_{0},c_{1}\right)=\left(1/\sqrt{2},1/\sqrt{2}\right)$ in (7). With the selection parameter $\gamma_{1}$ set at 1.5, we sought for $\gamma_{0}$ satisfying the $1/\tilde{S}\sum_{s=1}^{\tilde{S}}P\left(\text{select}|n_{i1}^{\left(s\right)},n_{i0}^{\left(s\right)}\right)=p$ as $P\left(\text{select}\right)=\int P\left(\text{select}|n_{i1}^{\left(s\right)},n_{i0}^{\left(s\right)}\right)f_{P}\left(n_{i1}^{\left(s\right)},n_{i0}^{\left(s\right)}\right)dn_{i1}^{\left(s\right)}dn_{i0}^{\left(s\right)}$, given the fixed marginal publication probability p as 0.7, indicating that approximately 70% of studies would be selectively published. To simulate the selective publication process, we decided whether an individual study would be published or not by a Bernoulli distribution, $\text{Bern}\left(\Phi \left(\gamma_{0}+\gamma_{1}t^{\left(s\right)}\right)\right)$.

To confirm whether the generated dataset had sparsity, we evaluated the proportion of the studies with zero entries in the 2 × 2 contingency table among all the studies (published and unpublished), as well as among the published studies. In addition, we evaluated the proportion of the studies with cell frequencies of no more than 3 and no more than 5. Their averages over the 1000 simulated datasets are summarized in Table 3. It indicates that we successfully generated meta‐analyses of sparsity.

**TABLE 3:** Summary of the sparsity of the simulated datasets.

		S = 15		S = 25		S = 50		S = 100	
Experiment	Rate (%)	Full	Published	Full	Published	Full	Published	Full	Published
1	Zero entries	21.0	19.7	20.9	19.7	20.8	19.3	21.5	20.2
	No‐more‐than‐3‐entries	80.5	75.4	80.4	74.9	80.8	75.2	81.0	75.8
	No‐more‐than‐5‐entries	93.4	90.1	93.3	90.0	93.8	90.2	93.5	90.2
2	Zero entries	0.6	0.9	0.6	0.9	0.7	1.0	0.8	1.1
	No‐more‐than‐3‐entries	15.0	18.3	14.9	18.6	15.1	18.4	15.7	18.4
	No‐more‐than‐5‐entries	38.4	43.1	38.2	43.5	38.4	43.2	38.8	42.9
3	Zero entries	5.4	6.7	5.5	6.9	5.3	6.7	5.0	6.5
	No‐more‐than‐3‐entries	50.0	47.8	49.4	46.9	49.5	47.0	48.2	46.0
	No‐more‐than‐5‐entries	73.6	70.7	73.3	70.2	73.5	70.3	72.9	70.2
4	Zero entries	15.5	23.2	15.2	23.3	15.5	23.3	14.9	22.7
	No‐more‐than‐3‐entries	63.6	70.5	63.2	70.7	63.6	70.9	64.2	71.3
	No‐more‐than‐5‐entries	80.2	84.7	80.3	84.9	80.7	85.1	81.6	85.8
5	Zero entries	1.4	3.6	1.3	3.7	1.4	3.6	1.3	4.1
	No‐more‐than‐3‐entries	21.4	30.5	21.5	30.4	21.1	30.1	21.5	30.9
	No‐more‐than‐5‐entries	45.9	54.4	45.9	54.1	44.9	53.5	45.3	54.0
6	Zero entries	4.1	10.7	4.0	11.1	3.4	10.8	3.3	10.9
	No‐more‐than‐3‐entries	40.0	50.7	40.7	51.5	39.2	50.7	40.5	51.6
No‐more‐than‐5‐entries	64.4	71.8	64.5	72.2	63.7	71.6	64.0	71.7

*Note:* Full indicates published and unpublished studies; Published indicates published studies.

We applied our proposed method to only published studies and compared our proposal with the sensitivity analysis proposed by Zhou et al. [12] based on the bivariate normal model. Specifying p=0.7 and the *t*‐statistics with $\left(c_{0},c_{1}\right)=\left(1/\sqrt{2},1/\sqrt{2}\right)$ in (7), we summarized the estimates for parameters in the model and the SAUC in Table 4. The average for the SAUC by maximizing the likelihood with the bivariate binomial model with only published studies (denoted as the MLE with published studies) had a non‐ignorable discrepancy with the true value, indicating that PB was a considerable issue for this dataset. Both the proposed method and the method by Zhou et al. [12] successfully reduced biases. Our proposed method had smaller biases than the method by Zhou et al. [12]. In addition, the empirical standard error, which is the standard deviation of point estimators for 1000 independent meta‐analyses [19], of our proposed method continued to decrease as the meta‐analysis size $\tilde{S}$ increased, further validating the superior performance of our proposed method. We also showed the estimated SAUC with two misspecified *t*‐statistics in the selection function (7) corresponding to $\left(c_{0},c_{1}\right)=\left(0,1\right)$ and (1,0) in Table 4. We observed that the misspecification of the *t*‐statistics in the selection function could lead to a slightly larger bias compared with that under the correctly specified *t*‐statistics. Correct specification of the *t*‐statistics in the selection function could remove the bias. Except for the estimates of SAUC, we showed the estimates of (θ,α) in the bivariate binomial model and the pairs of sensitivity and specificity in Web Table 6. It substantiated that our proposed method could also obtain the least bias for these parameters and statistics among the three methods.

**TABLE 4:** Summary of the SAUC estimates under the true selection mechanism of $\left(c_{0},c_{1}\right)=\left(1/\sqrt{2},1/\sqrt{2}\right)$ given p≈0.7.

			S = 15	S = 25	S = 50	S = 100
Experiment	Method	TRUE	AVE (SD)	AVE (SD)	AVE (SD)	AVE (SD)
1	MLE with published studies	83.2	87.5 (4.4)	87.4 (3.5)	87.6 (2.5)	87.5 (1.7)
	Method of Zhou et al. [12]		84.8 (4.3)	84.8 (3.3)	85.1 (2.4)	85.1 (1.7)
	Proposal with $\left(c_{0},c_{1}\right)=\left(1/\sqrt{2},1/\sqrt{2}\right)$		84.4 (5.8)	83.9 (4.7)	83.7 (3.4)	83.6 (2.5)
	Proposal with $\left(c_{0},c_{1}\right)=\left(1,0\right)$		86.3 (4.9)	86.3 (3.9)	86.6 (2.8)	86.4 (1.9)
	Proposal with $\left(c_{0},c_{1}\right)=\left(0,1\right)$		86.9 (4.8)	87.4 (3.7)	87.9 (2.6)	87.3 (1.8)
2	MLE with published studies	79.8	81.7 (7.4)	82.7 (5.2)	83.8 (3.7)	84.4 (2.7)
	Method of Zhou et al. [12]		76.2 (9.4)	76.8 (7.0)	78.0 (5.1)	78.3 (3.9)
	Proposal with $\left(c_{0},c_{1}\right)=\left(1/\sqrt{2},1/\sqrt{2}\right)$		79.1 (8.5)	79.4 (6.6)	80.1 (5.0)	80.5 (3.9)
	Proposal with $\left(c_{0},c_{1}\right)=\left(1,0\right)$		82.0 (7.4)	82.7 (5.1)	83.7 (3.6)	84.2 (2.8)
	Proposal with $\left(c_{0},c_{1}\right)=\left(0,1\right)$		78.6 (7.7)	78.6 (5.6)	79.0 (4.0)	79.4 (2.9)
3	MLE with published studies	86.9	88.8 (3.1)	89.3 (2.4)	89.7 (1.5)	89.7 (1.0)
	Method of Zhou et al. [12]		84.6 (4.3)	85.0 (3.6)	85.6 (2.5)	85.7 (1.9)
	Proposal with $\left(c_{0},c_{1}\right)=\left(1/\sqrt{2},1/\sqrt{2}\right)$		87.8 (4.1)	88.1 (3.4)	88.2 (2.5)	88.0 (1.9)
	Proposal with $\left(c_{0},c_{1}\right)=\left(1,0\right)$		89.4 (3.5)	90.1 (2.6)	90.7 (1.7)	91.0 (1.1)
Proposal with $\left(c_{0},c_{1}\right)=\left(0,1\right)$	87.5 (3.3)		87.7 (2.5)	87.9 (1.7)	87.8 (1.1)
4	MLE with published studies	83.2	84.0 (3.1)	84.1 (2.2)	84.3 (1.5)	84.3 (1.0)
	Method of Zhou et al. [12]		81.2 (3.3)	81.1 (2.3)	81.1 (1.6)	81.1 (1.1)
	Proposal with $\left(c_{0},c_{1}\right)=\left(1/\sqrt{2},1/\sqrt{2}\right)$		83.0 (3.8)	83.1 (2.8)	83.3 (2.0)	83.3 (1.3)
	Proposal with $\left(c_{0},c_{1}\right)=\left(1,0\right)$		83.9 (3.3)	84.0 (2.3)	84.2 (1.6)	84.2 (1.1)
	Proposal with $\left(c_{0},c_{1}\right)=\left(0,1\right)$		84.0 (3.2)	84.1 (2.2)	84.2 (1.5)	84.3 (1.1)
5	MLE with published studies	79.8	80.9 (4.3)	81.4 (3.0)	81.4 (2.2)	81.6 (1.5)
	Method of Zhou et al. [12]		77.3 (5.5)	77.6 (3.9)	77.4 (2.8)	77.4 (2.1)
	Proposal with $\left(c_{0},c_{1}\right)=\left(1/\sqrt{2},1/\sqrt{2}\right)$		79.6 (5.7)	80.3 (4.3)	80.2 (3.4)	80.3 (2.6)
	Proposal with $\left(c_{0},c_{1}\right)=\left(1,0\right)$		81.0 (4.5)	81.5 (3.2)	81.4 (2.3)	81.4 (1.6)
	Proposal with $\left(c_{0},c_{1}\right)=\left(0,1\right)$		80.1 (4.6)	80.6 (3.1)	80.6 (2.3)	80.7 (1.6)
6	MLE with published studies	86.9	87.3 (2.3)	87.5 (1.9)	87.7 (1.3)	87.8 (0.8)
	Method of Zhou et al. [12]		84.0 (3.3)	84.3 (2.5)	84.4 (1.7)	84.5 (1.1)
	Proposal with $\left(c_{0},c_{1}\right)=\left(1/\sqrt{2},1/\sqrt{2}\right)$		87.0 (3.1)	87.5 (2.7)	87.8 (2.1)	88.2 (1.2)
	Proposal with $\left(c_{0},c_{1}\right)=\left(1,0\right)$		87.8 (2.5)	88.2 (2.1)	88.5 (1.6)	88.9 (1.2)
	Proposal with $\left(c_{0},c_{1}\right)=\left(0,1\right)$		86.9 (2.4)	87.1 (2.0)	87.2 (1.4)	87.4 (0.8)

*Note:* The estimates are summarized by mean (empirical standard error) over 1000 simulated meta‐analyses; the values are multiplied by 100.

We show the results with simulation datasets under the true selective mechanisms with $\left(c_{0},c_{1}\right)=\left(0,1\right)$ and (1,0) in Web Appendix G.

To validate the robustness under various marginal selection probabilities p, we conducted additional simulation studies with the same setups as the one in this section but changing the marginal selection probability p from 0.7 to 0.5. It reflects a heavier missing publication case where around 50% of studies are supposed to be unpublished. We showed the summary results in Web Appendix H. The average of SAUCs under our proposed method was closer to the true values compared with the method by Zhou et al. [12], suggesting the advantage of the bivariate binomial model in sparse meta‐analysis. In addition, the estimate by our proposed method in Table 4 and Web Table 12 are very close, indicating the robustness of our proposed method across various marginal selection probabilities. In addition, we compared the proposed method with existing ones when the marginal selection probability was misspecified. We applied the proposed and related methods assuming p=0.5 to the simulation datasets, which had p=0.7. The results were summarized in Web Appendix I. We found that even though the SAUC estimated by our proposed method contained unignorable bias under misspecified p, they were still less biased compared to the method by Zhou et al. [12] in most scenarios, indicating the advantage of our proposed method in sparse diagnostic meta‐analyses.

## 6 Discussion

We proposed an exact likelihood‐based sensitivity analysis method to address PB in meta‐analyses of diagnostic studies with sparse data. Most existing sensitivity analysis methods rely on the normal approximation for the pair of empirical logit‐transformed sensitivities and specificities, while we utilize the bivariate binomial model that uses the exact within‐study binomial model. The bivariate binomial model is more suitable for sparse meta‐analyses and possesses better finite sample performance. To our knowledge, Hattori and Zhou [13] is the only sensitivity analysis method for PB in the bivariate binomial model. They used the Heckman‐type selection function. Their method described a selective publication process under which studies of larger sample sizes and larger AUC were more likely to be published. On the other hand, the method for the SROC was designed to make inferences based on the observations of pairs of sensitivity and specificity, and some studies might not report the AUC. Thus, the publication of such studies would be determined by sensitivity and specificity rather than the AUC. To address this issue, we extended the Copas *t*‐statistics selection model to the bivariate binomial model. Our selection function defines selective publication processes by the observed statistics of each study and its cut‐off value. Such selective publication based on observed quantities would be more appealing. Since the true structure of the selective publication is unknown, we cannot determine which selection model would be more reliable between the Copas‐Heckman and Copas *t*‐statistics selection models. It is important to evaluate robustness against various kinds of potential selective publication processes. Our development provides a useful tool to evaluate the potential impacts of selective publication processes alternative to the Copas‐Heckman selection model. The results of the real applications and simulation studies showed the feasibility of our proposal in practical situations, especially for sparse meta‐analyses of diagnostic studies. We consider the selection function as a monotone function with the *t*‐statistics of the linear combination of logit‐transformed sensitivity and specificity. Our methods can reflect several publication mechanisms including those determined by lnDOR, sensitivity, and specificity. However, the true underlying publication mechanism is not easy to identify or even verify with limited information from only published studies. Thus, we recommend conducting a comprehensive sensitivity analysis with multiple selection functions corresponding to various *t*‐statistics or $\left(c_{0},c_{1}\right)$ in practice. In addition, Zhou et al. [12] proposed a joint estimation of model parameters and $\left(c_{0},c_{1}\right)$ in the selection model for the bivariate normal model. We also tried to assume $\left(c_{0},c_{1}\right)$ as parameters to estimate rather than hyperparameters and jointly estimate them with other model parameters in the bivariate binomial model. We found the optimization was not stable and often fell into non‐convergence. It is appealing to develop a more robust estimation method to overcome the computational issue so that the $\left(c_{0},c_{1}\right)$ can be estimated for sparse meta‐analyses in the future.

Though the use of the bivariate binomial model can avoid continuous correction when a zero cell occurs, we have to apply continuous correction to compute *t*‐statistics in the selection model. For those studies with zero cells, the sensitivity/specificity/DOR is undefined, thus, the continuity correction is unavoidable to describe the selective mechanism. Thus, from this perspective, our proposal has reduced the effect of continuity correction to a minimum. However, the use of continuity correction will still lead to inductive bias in the results especially in meta‐analysis with a large proportion of studies containing zero cells in the selection function. It is warranted in future research to define relevant selection functions completely free from influential continuity correction.

We took a sensitivity analysis approach with the marginal selection probability p fixed. In reality, p is unknown. Thus, we need to consider several values of the marginal selection probability. Even with this difficulty, which is a common issue in the sensitivity analysis, our simulation study revealed that the proposed method is better suited for sparse meta‐analysis. Li et al. [17] proposed EM algorithms to quantitatively estimate the parameters in the bivariate normal model and the SROC curve/SAUC, adjusting for PB without assuming a given selection probability. Their methods are based on the Copas‐Heckman selection model. It would be interesting to develop methods to adjust for PB in sparse meta‐analysis of diagnostic studies with the Copas *t*‐statistics selection functions, without specifying the sensitivity parameters and the marginal selection probability. Moreover, in this paper, we modeled the selection function only with *t*‐statistics. Other study characteristics can influence publication. It would be very important to investigate how to handle the covariate‐specific selection function in future work.

## Funding

This work was supported by Grant‐in‐Aid for Scientific Research, 25K03086 from the Ministry of Education, Science, Sports and Technology of Japan.

## Conflicts of Interest

The authors declare no conflicts of interest.

## Supporting information


**Data S1:** Supporting Information.

## Data Availability

Data and codes in the form of R together with complete documentation is available on GitHub at https://github.com/Taojun‐Hu/meta‐analysis‐pb‐diagnostic.
