# Supplementary material for: Sensitivity Analysis for Publication Bias in Diagnostic Meta‐Analysis of Sparsity Using the Copas t‐Statistic Selection Function
Source: Stat Med. 2026 Mar 18;45(6-7):e70465. doi: 10.1002/sim.70465 (PMC12997088; doi:10.1002/sim.70465)
Supplement: Supplementary file 1 — Data S1: Supporting Information. [file SIM-45-0-s001.pdf]

Received: Added at production | Revised: Added at production | Accepted: Added at production

DOI: xxx/xxxx

SUPPLEMENTARY MATERIAL

Supplementary Materials for " Sensitivity analysis for publication bias in diagnostic meta-analysis of sparsity using the Copas t-statistic selection function " by Taojun Hu, Yi Zhou, Xiao-Hua Zhou and Satoshi Hattori

Taojun Hu[1,2] | Yi Zhou[3,1] | Xiao-Hua Zhou[2,4] | Satoshi Hattori[1,5]

[1]Department of Biomedical Statistics, Graduate School of Medicine, Osaka University, Osaka, Japan

[2]Department of Biostatistics, School of Public Health, Peking University, Beijing, China

[3]Division of Mathematics and Informatics, Graduate School of Human Development and Environment, Kobe University, Kobe, Japan

[4]Beijing International Center for Mathematical Research, Peking University, Beijing, China

[5]Integrated Frontier Research for Medical Science Division, Institute for Open and Transdisciplinary Research Initiatives (OTRI), Osaka University, Osaka, Japan

Correspondence
Corresponding author: Satoshi Hattori
Email: hattoris@biostat.med.osaka-u.ac.jp;
Co-corresponding author: Xiao-Hua Zhou
Email: azhou@math.pku.edu.cn

Abstract

KEYWORDS

Web Appendix A | METHODS ADDRESSING PUBLICATION BIAS IN UNIVARIATE AND DIAGNOSTIC META-ANALYSIS

In univariate meta-analysis, graphical methods like the funnel-plot and trim-and-fill methods [1,2] presented accessible ways to detect and adjust for PB. However, graphical methods could be subjective and less informative for the selection mechanism of studies. In contrast to the graphical methods, the sensitivity analysis method models the mechanism of selective publication using the selection function and would give us a more insightful interpretation of publication bias (PB). Copas and colleagues [3,4,5] first introduced a selection model based on the Heckman model [6,7] and assumed that whether a study would be published or not was determined by a latent Gaussian random variable. We refer to this sensitivity analysis method proposed by Copas and Shi [5] as the Copas-Heckman selection model. Modeling with a latent Gaussian random variable is convenient to handle the normal-normal (NN) random-effects model for the outcome; the likelihood conditional on the published can be easily derived based on the joint normal distribution. On the other hand, since the selective publication is described through a latent variable, it is not necessarily easy to clarify what kind of selective publication is behind the meta-analysis. Copas [8] introduced an alternative way for sensitivity analysis to allow selection functions monotonic with the t-statistic, or equivalently its p-value, given in each

 © 2023 Copyright Holder Name

individual study. We call it the Copas t-statistic selection model. This model relies on fewer sensitivity parameters and is more interpretable than the Copas-Heckman selection model[8].

Compared with the development of the methods against PB for univariate meta-analysis as reviewed, methods for addressing PB in meta-analysis of diagnostic studies remain understudied. Simple methods based on funnel-plot asymmetry such as Begg, Egger, and Macaskill tests[9,10,11] have been applied to some univariate diagnosis measures such as the log diagnostic odds ratio (lnDOR)[12,13]. Lin et al.[14] compared these tests with empirical studies. Recently, there have been some studies working on proposing novel tests or PB-adjusted methods for diagnostic meta-analysis. Hong et al.[15] proposed a test of PB for multivariate meta-analysis including diagnostic meta-analysis. Luo et al.[16] proposed to adjust for PB in diagnostic meta-analysis using a bivariate trim-and-fill approach. However, none of them can directly address the impact of selective publication on the SROC curve. Consequently, developing methods to address PB on the SROC curve/SAUC is more attractive. Piao et al.[17] devised a likelihood-based method for parameter estimation with the Copas-Heckman selection model, applied to the bivariate normal model using an EM algorithm. Li et al.[18] took a different approach to correct PB in the bivariate normal model using empirical likelihood, also relying on the Copas-Heckman selection model. A versatile sensitivity analysis technique with the bivariate normal model utilizing the Copas t-statistics selection model was proposed by Zhou et al.[19]. This approach has shown considerable promise in addressing PB using the Copas t-statistics selection model in meta-analysis of diagnostic studies. Zhou et al.[20] further proposed a nonparametric worst-case bound over selection models that are monotone with respect to variance. Though the nonparametric worst-case bounds restrict less on the selection models, the bounds can be very large and less interpretable. Meanwhile, it is very difficult to derive the confidence intervals for the nonparametric bounds. However, all the above methods are for the bivariate normal model[21]. Regarding the bivariate binomial model, only Hattori and Zhou[22] introduced a sensitivity analysis method based on the Copas-Heckman selection model. This extended Copas-Heckman selection model describes a selective publication process in which studies with larger AUCs are more likely to be published. Since each individual study does not report the ROC and AUC, this modeling of the selective publication is not necessarily appealing. In contrast, the Copas t-statistic model is more interpretable than the Copas-Heckman selection when addressing PB in practice; one can model the selective publication process with the t-statistics of the observed quantities in the paper of each individual study, such as sensitivity and specificity. To our knowledge, the only extension of the Copas t-statistics selection model for PB in meta-analysis of diagnostic studies is based on the bivariate normal model[19]. To make up for the lack of methods for addressing PB in meta-analysis with sparse data of diagnostic studies, it is necessary to extend the Copas t-statistics selection model to the bivariate binomial model. Note that all the above methods to address PB on SROC/SAUC are based on selection models. To ease readers' better comparison of these existing methods and our proposed method, we present the pros and cons of the existing selection-based methods and our proposal for addressing PB on SROC/SAUC in Web Table 1.

Web Appendix B | AN INTRODUCTION TO THE METHOD OF ZHOU ET AL.[19]

Since the Method of Zhou et al.[19] is the main method we compare our proposed method with, we introduce how their method is performed in detail in this section.

Each study i ($i = 1, 2, \ldots, S$) provides the observed counts of true positives, false negatives, true negatives, and false positives, respectively denoted as $n^i_{11}, n^i_{01}, n^i_{00}$, and n^i_{10}. Let $n^i_{+1} = n^i_{11} + n^i_{01}$ represent the total number of diseased individuals and $n^i_{+0} = n^i_{00} + n^i_{10}$ the total number of non-diseased individuals in study i. The observed sensitivity and specificity for each study are calculated as $\widehat{\text{se}}_i = n^i_{11}/n^i_{+1}$ and $\widehat{\text{sp}}_i = n^i_{00}/n^i_{+0}$, respectively. Zhou et al.[19] employ a bivariate normal model[21] to model sensitivity and specificity, defining μ_{1i} and μ_{2i} as the logit-transformed values of the true sensitivity and true specificity for the ith study. The bivariate normal model posits that $(\mu_{1i}, \mu_{2i})^T$ follows a normal distribution:

$$\begin{pmatrix}\mu_{1i}\\ \mu_{2i}\end{pmatrix} \sim N\left(\begin{pmatrix}\mu_1\\ \mu_2\end{pmatrix}, \mathbf{\Omega}\right) \text{ with } \mathbf{\Omega} = \begin{pmatrix}\tau_1^2 & \tau_{12}\\ \tau_{12} & \tau_2^2\end{pmatrix}, \tag{S.1}$$

where μ_1 and μ_2 denote the overall mean values of the logit-transformed sensitivity and logit-transformed specificity, τ_1^2 ($\tau_1 > 0$) and τ_2^2 ($\tau_2 > 0$) are the between-study variances for these two transformed metrics, $\tau_{12} = \rho\tau_1\tau_2$ is the covariance of μ_{1i} and μ_{2i}, and ρ ($-1 \le \rho \le 1$) is the corresponding correlation coefficient. Let y_{1i} and y_{2i} represent the logit-transformed versions of

WEB TABLE 1 The pros and cons of the existing selection-function-based methods in addressing PB on SROC/SAUC

Category	Model for the outcome	Selection function	Methods	Pros	Cons
PB-corrected estimation	Bivariate normal	Copas-Heckman	Piao et al.[17]; Li et al.[18]	Directly correct PB without specifying sensitivity parameters.	Rely on the asymptotic normality of the outcome of each study. Estimations can be unstable since relying on the conditional likelihood given published.
Sensitivity analysis	Bivariate normal	Copas-Heckman	Not found	-	-
		Copas t-statistics	Zhou et al.[19]	Selection models of more interpretability with observed test statistics.	Rely on the asymptotic normality of the outcome of each study.
	Bivariate binomial	Copas-Heckman	Hattori and Zhou[22]	AUC-dependent selective publication.	AUC of each study is latent.
		Copas t-statistics	Our proposal	Selection models of more interpretability with observed test statistics. Does not rely on the normal approximation of the outcome of each study and is stable for sparse data.	Computationally demanding compared to the binomial normal model.
Nonparametric worst-case bound	Bivariate normal	Monotone with respect to variance	Zhou et al.[20]	Less restrictions on selection models.	Bound can be very large of less interpretability. Confidence intervals cannot be given.

the observed sensitivity ($\widehat{\text{se}}_i$) and observed specificity ($\widehat{\text{sp}}_i$), respectively. Conditional on (μ_{1i}, μ_{2i}), the model assumes that:

$$\begin{pmatrix} y_{1i} \\ y_{2i} \end{pmatrix} \sim N\left(\begin{pmatrix} \mu_{1i} \\ \mu_{2i} \end{pmatrix}, \mathbf{\Sigma}_i \right) \text{ with } \mathbf{\Sigma}_i = \begin{pmatrix} s_{1i}^2 & 0 \\ 0 & s_{2i}^2 \end{pmatrix}, \tag{S.2}$$

where s_{1i}^2 and s_{2i}^2 correspond to the observed within-study variances of y_{1i} and y_{2i}, respectively. For studies containing zero counts in any cell, a continuity correction is applied by adding 0.5 to each of the four cells $(n_{11}^i, n_{01}^i, n_{00}^i, n_{10}^i)$.

In line with standard practices for literature-based meta-analyses, Zhou et al.[19] treat $\mathbf{\Sigma}_i$ as a known quantity. Combining the models in (S.1) and (S.2) yields the following marginal model:

$$\boldsymbol{y}_i \mid \mathbf{\Sigma}_i \sim N_2\left(\boldsymbol{\mu}, \mathbf{\Omega} + \mathbf{\Sigma}_i\right), \tag{S.3}$$

where $\boldsymbol{y}_i = (y_{1i}, y_{2i})^T$, $\boldsymbol{\mu} = (\mu_1, \mu_2)^T$, and N_2 denotes the bivariate normal distribution. Zhou et al.[19] further propose a more general form of selection function, defined on a t-type statistic derived from a linear combination of the logit-transformed sensitivity and logit-transformed specificity:

$$\boldsymbol{c}^T\boldsymbol{y}_i = c_0 y_{1i} + c_1 y_{2i},$$

where $\boldsymbol{c} = (c_0, c_1)^T$ denotes a contrast vector. Using the marginal distribution in (S.3), the following relationship holds:

$$t_i = \frac{\boldsymbol{c}^T\boldsymbol{y}_i}{\sqrt{\boldsymbol{c}^T\mathbf{\Sigma}_i\boldsymbol{c}}} \sim N\left(\frac{\boldsymbol{c}^T\boldsymbol{\mu}}{\sqrt{\boldsymbol{c}^T\mathbf{\Sigma}_i\boldsymbol{c}}}, 1 + \frac{\boldsymbol{c}^T\mathbf{\Omega}\boldsymbol{c}}{\boldsymbol{c}^T\mathbf{\Sigma}_i\boldsymbol{c}} \right). \tag{S.4}$$

Owing to the scale invariance of the t-type statistic, Zhou et al.[19] impose the constraint $\boldsymbol{c}^T\boldsymbol{c} = 1$ $(0 \le c_0, c_1 \le 1)$ without loss of generality. For instance, setting $(c_0, c_1) = (1/\sqrt{2}, 1/\sqrt{2})$ results in the t-statistic corresponding to the natural logarithm of the diagnostic odds ratio (lnDOR). Zhou et al.[19] then define the selection function $P(\text{select} \mid \boldsymbol{y}_i, \mathbf{\Sigma}_i)$ as a function a that

depends on t_i:

$$P(\text{select} \mid \boldsymbol{y}_i, \boldsymbol{\Sigma}_i) = a(\boldsymbol{y}_i, \boldsymbol{\Sigma}_i) = a(t_i). \tag{S.5}$$

Zhou et al.[19] adopt the probit function following Copas[8]:

$$P(\text{select} \mid \boldsymbol{y}_i, \boldsymbol{\Sigma}_i) = a(t_i) = \Phi(\beta t_i + \alpha), \tag{S.6}$$

where β and α are parameters that regulate the probability of selective publication.

Let $p = P(\text{select})$ denote the marginal probability of a study being selected for publication. Using the selection function defined in (S.5), we have:

$$p = P(\text{select}) = E_P\{a(t_i)\},$$

where this expression represents the expected proportion of studies from the underlying population that are published and included in the meta-analysis. By leveraging the definition of the probit function, (S.6) can be re-expressed as:

$$P(\text{select} \mid \boldsymbol{y}_i, \boldsymbol{\Sigma}_i) = a(t_i) = P(z_i < \beta t_i + \alpha \mid \boldsymbol{y}_i, \boldsymbol{\Sigma}_i) = \Phi\left(\beta \frac{\boldsymbol{c}^T \boldsymbol{y}_i}{\sqrt{\boldsymbol{c}^T \boldsymbol{\Sigma}_i \boldsymbol{c}}} + \alpha\right), \tag{S.7}$$

where z_i follows a standard normal distribution ($z_i \sim N(0,1)$) and is independent of t_i.

By substituting the distribution of t_i from (S.4) into the selection function $a(t_i)$, we derive the function $b(\boldsymbol{\Sigma}_i)$:

$$P(\text{select} \mid \boldsymbol{\Sigma}_i) = b(\boldsymbol{\Sigma}_i) = P\left(z_i - \beta t_i < \alpha \mid \boldsymbol{\Sigma}_i\right) = \Phi\left\{\frac{\beta \dfrac{\boldsymbol{c}^T \boldsymbol{\mu}}{\sqrt{\boldsymbol{c}^T \boldsymbol{\Sigma}_i \boldsymbol{c}}} + \alpha}{\sqrt{1 + \beta^2 \left(1 + \dfrac{\boldsymbol{c}^T \boldsymbol{\Omega} \boldsymbol{c}}{\boldsymbol{c}^T \boldsymbol{\Sigma}_i \boldsymbol{c}}\right)}}\right\}.$$

Zhou et al.[19] use f_O to represent the distribution of quantities across the S published (observed) studies, and f_P to represent the distribution across the S studies in the full population (encompassing both published and unpublished studies).

For a specified marginal publication selection probability $p = P(\text{select})$, the distribution of the observed $\boldsymbol{\Sigma}_i$ is given by:

$$f_O(\boldsymbol{\Sigma}_i) = P(\boldsymbol{\Sigma}_i \mid \text{select}) = \frac{P(\text{select} \mid \boldsymbol{\Sigma}_i) P(\boldsymbol{\Sigma}_i)}{P(\text{select})} = \frac{b(\boldsymbol{\Sigma}_i) f_P(\boldsymbol{\Sigma}_i)}{p},$$

and rearranging this expression yields:

$$f_P(\boldsymbol{\Sigma}_i) = p \frac{1}{b(\boldsymbol{\Sigma}_i)} f_O(\boldsymbol{\Sigma}_i). \tag{S.8}$$

Integrating both sides of (S.8) with respect to $\boldsymbol{\Sigma}_i$ results in:

$$p = [E_O\{b(\boldsymbol{\Sigma}_i)^{-1}\}]^{-1}, \tag{S.9}$$

with an approximate relationship given by $p \approx S / \sum_{i=1}^{S} \{b(\boldsymbol{\Sigma}_i)^{-1}\}$. Lastly, the joint distribution of the observed pairs $(\boldsymbol{y}_i, \boldsymbol{\Sigma}_i)$ is expressed as:

$$\begin{aligned}
f_O(\boldsymbol{y}_i, \boldsymbol{\Sigma}_i) &= P(\boldsymbol{y}_i, \boldsymbol{\Sigma}_i \mid \text{select}) \\
&= \frac{f_P(\boldsymbol{y}_i, \boldsymbol{\Sigma}_i) a(\boldsymbol{y}_i, \boldsymbol{\Sigma}_i)}{p} \\
&= \frac{f_P(\boldsymbol{y}_i \mid \boldsymbol{\Sigma}_i) f_P(\boldsymbol{\Sigma}_i) a(\boldsymbol{y}_i, \boldsymbol{\Sigma}_i) f_O(\boldsymbol{\Sigma}_i)}{b(\boldsymbol{\Sigma}_i) f_P(\boldsymbol{\Sigma}_i)} \\
&= \frac{f_P(\boldsymbol{y}_i \mid \boldsymbol{\Sigma}_i) a(\boldsymbol{y}_i, \boldsymbol{\Sigma}_i) f_O(\boldsymbol{\Sigma}_i)}{b(\boldsymbol{\Sigma}_i)},
\end{aligned}$$

which leads to the following log-likelihood function for the published studies:

$$\begin{aligned}\ell_O(\boldsymbol{\mu}, \boldsymbol{\Omega}, \boldsymbol{c}, \beta, \alpha) &= \log \prod_{i=1}^{S} f_O(\boldsymbol{y}_i, \boldsymbol{\Sigma}_i) \\ &= \sum_{i=1}^{S} \log f_P(\boldsymbol{y}_i \mid \boldsymbol{\Sigma}_i) + \sum_{i=1}^{S} \log a(\boldsymbol{y}_i, \boldsymbol{\Sigma}_i) - \sum_{i=1}^{S} \log b(\boldsymbol{\Sigma}_i) + \sum_{i=1}^{S} \log f_O(\boldsymbol{\Sigma}_i). \end{aligned} \tag{S.10}$$

The first term in (S.10) corresponds to the log-likelihood of the bivariate normal model when publication bias is not considered. The second and third terms collectively account for the correction of publication bias. The final term is a constant that depends solely on the observed variances, and thus does not influence the maximization of ℓ_O.

For statistical inference, Zhou et al.[19] fix the marginal publication selection probability p as a sensitivity parameter. Recall that (S.9) provides the approximate relationship $p \approx S / \sum_{i=1}^{S} \{b(\boldsymbol{\Sigma}_i)^{-1}\}$; solving this equation for α expresses α as a function of the remaining parameters $(\boldsymbol{\mu}, \boldsymbol{\Omega}, \boldsymbol{c}, \beta)$ and the specified p. Zhou et al.[19] denote this function as $\alpha_p = \alpha_p(\boldsymbol{\mu}, \boldsymbol{\Omega}, \boldsymbol{c}, \beta)$. Ultimately, for a given p, the log-likelihood in (S.10) can be re-expressed as a function of $(\boldsymbol{\mu}, \boldsymbol{\Omega}, \boldsymbol{c}, \beta)$:

$$\begin{aligned}\ell_O(\boldsymbol{\mu}, \boldsymbol{\Omega}, \boldsymbol{c}, \beta) &= \ell_O(\boldsymbol{\mu}, \boldsymbol{\Omega}, \boldsymbol{c}, \beta, \alpha_p) \\ &= \sum_{i=1}^{S} \left\{ -\frac{1}{2}(\boldsymbol{y}_i - \boldsymbol{\mu})^T (\boldsymbol{\Sigma}_i + \boldsymbol{\Omega})^{-1} (\boldsymbol{y}_i - \boldsymbol{\mu}) - \frac{1}{2} \log \mid \boldsymbol{\Sigma}_i + \boldsymbol{\Omega} \mid \right\} \\ &+ \sum_{i=1}^{S} \log \Phi \left(\beta \frac{\boldsymbol{c}^T \boldsymbol{y}_i}{\sqrt{\boldsymbol{c}^T \boldsymbol{\Sigma}_i \boldsymbol{c}}} + \alpha_p \right) - \sum_{i=1}^{S} \log \Phi \left\{ \frac{\beta \frac{\boldsymbol{c}^T \boldsymbol{\mu}}{\sqrt{\boldsymbol{c}^T \boldsymbol{\Sigma}_i \boldsymbol{c}}} + \alpha_p}{\sqrt{1 + \beta^2 \left(1 + \frac{\boldsymbol{c}^T \boldsymbol{\Omega} \boldsymbol{c}}{\boldsymbol{c}^T \boldsymbol{\Sigma}_i \boldsymbol{c}} \right)}} \right\}. \end{aligned} \tag{S.11}$$

Parameter estimates are obtained by maximizing this conditional log-likelihood function (S.11).

Web Appendix C | APPROXIMATION METHODS TO CALCULATE $\mathrm{P}(\mathrm{SELECT} \mid N_1^{(S)}, N_0^{(S)})$

As mentioned in Section 3 of our main body, to handle the computation of $\mathrm{P}\left(\text{select} \mid n_1^{(s)}, n_0^{(s)}\right)$ for each study, we proposed an approximation method based on the asymptotic normal distribution of $t\left(m_{11}, n_1^{(s)} - m_{11}, n_0^{(s)} - m_{00}, m_{00}\right)$ (see Equation (15) in the main body), which is given by

$$\frac{c_1 \log \left(\frac{m_{11}/n_1^{(s)}}{1 - m_{11}/n_1^{(s)}} \right) + c_0 \log \left(\frac{m_{00}/n_0^{(s)}}{1 - m_{00}/n_0^{(s)}} \right)}{\sqrt{c_1^2 / \left(n_0^{(s)} \frac{m_{00}}{n_0^{(s)}} (1 - \frac{m_{00}}{n_0^{(s)}}) \right) + c_0^2 / \left(n_1^{(s)} \frac{m_{11}}{n_1^{(s)}} (1 - \frac{m_{11}}{n_1^{(s)}}) \right)}}. \tag{S.12}$$

For simplification of notations, we let $\tilde{t}^{(s)} = t\left(m_{11}, n_1^{(s)} - m_{11}, n_0^{(s)} - m_{00}, m_{00}\right)$.

It could be separated into two parts. Let $\widehat{p}_1^{(s)} = m_{11}/n_1^{(s)}$, and $\widehat{p}_0^{(s)} = m_{00}/n_0^{(s)}$, $M_1^{(s)} = n_1^{(s)} \widehat{p}_1^{(s)} (1 - \widehat{p}_1^{(s)})$, and $M_0^{(s)} = n_0^{(s)} \widehat{p}_0^{(s)} (1 - \widehat{p}_0^{(s)})$. Let $S_1^{(s)} = \log \frac{\widehat{p}_1^{(s)}}{1 - \widehat{p}_1^{(s)}}$ and $S_0^{(s)} = \log \frac{\widehat{p}_0^{(s)}}{1 - \widehat{p}_0^{(s)}}$. Then, $\tilde{t}^{(s)}$ is simplified to

$$\frac{c_1 S_1^{(s)} + c_0 S_0^{(s)}}{\sqrt{c_1^2 / M_1^{(s)} + c_0^2 / M_0^{(s)}}} = \frac{S_1^{(s)}}{\sqrt{1/M_1^{(s)}}} \sqrt{\frac{c_1^2 / M_1^{(s)}}{c_1^2 / M_1^{(s)} + c_0^2 / M_0^{(s)}}} + \frac{S_0^{(s)}}{\sqrt{1/M_0^{(s)}}} \sqrt{\frac{c_0^2 / M_0^{(s)}}{c_1^2 / M_1^{(s)} + c_0^2 / M_0^{(s)}}} \tag{S.13}$$

For $\sqrt{\dfrac{c_1^2/M_1^{(s)}}{c_1^2/M_1^{(s)}+c_0^2/M_0^{(s)}}}$ in the first term of (S.13), we have

$$\sqrt{\frac{c_1^2/M_1^{(s)}}{c_1^2/M_1^{(s)}+c_0^2/M_0^{(s)}}}=\sqrt{\frac{1}{1+c_0^2/c_1^2\cdot M_1^{(s)}/M_0^{(s)}}}=\sqrt{\frac{1}{1+\dfrac{c_0^2}{c_1^2}\dfrac{n_1^{(s)}\widehat{p}_1^{(s)}(1-\widehat{p}_1^{(s)})}{n_0^{(s)}\widehat{p}_0^{(s)}(1-\widehat{p}_0^{(s)})}}}. \tag{S.14}$$

If $c_1=0$ or 1, this term reduces to 1 or 0. Thus, we only consider the situation when $c_1\in(0,1)$. We denote the sensitivity and specificity for the s-th study as $p_1^{(s)}$ and $p_0^{(s)}$, respectively. We regard m_{11} and m_{00} as the random variables which follow binomial distributions given $(p_1^{(s)},p_0^{(s)})$, $m_{11}\sim N(n_1^{(s)},p_1^{(s)})$ and $m_{00}\sim N(n_0^{(s)},p_0^{(s)})$. Under the large sample assumption, denoted as **Assumption (A.1)** $n_1^{(s)}\to+\infty$, and **Assumption (A.2)** $n_0^{(s)}\to+\infty$, then according to central limit theorems and the law of large numbers, we have $\widehat{p}_1^{(s)}\xrightarrow{p}p_1^{(s)},\widehat{p}_0^{(s)}\xrightarrow{p}p_0^{(s)}$, and

$$\begin{aligned}\sqrt{n_0^{(s)}}\left(\widehat{p}_1^{(s)}-p_0^{(s)}\right)&\xrightarrow{d}N(0,p_0^{(s)}(1-p_0^{(s)}))\\ \sqrt{n_1^{(s)}}\left(\widehat{p}_0^{(s)}-p_1^{(s)}\right)&\xrightarrow{d}N(0,p_1^{(s)}(1-p_1^{(s)})),\end{aligned} \tag{S.15}$$

where $\xrightarrow{d}$ implies the convergence in distribution. Additionally, we introduce another assumption:

Assumption (A.3): $\dfrac{n_1^{(s)}}{n_0^{(s)}}\xrightarrow{a.s.}K^{(s)}$.

This assumption posits that the proportions between the numbers of subjects with and without the disease converge to specific values. Then, based on this assumption, according to Slutsky's Theorem, we have

$$\frac{n_1^{(s)}\widehat{p}_1^{(s)}(1-\widehat{p}_1^{(s)})}{n_0^{(s)}\widehat{p}_0^{(s)}(1-\widehat{p}_0^{(s)})}\xrightarrow{p}K^{(s)}\frac{p_1^{(s)}(1-p_1^{(s)})}{p_0^{(s)}(1-p_0^{(s)})} \tag{S.16}$$

As $g(x)=\sqrt{\dfrac{1}{1+x}}$ is continuous, by the continuous mapping theorem, we have

$$\sqrt{\frac{c_1^2/M_1^{(s)}}{c_1^2/M_1^{(s)}+c_0^2/M_0^{(s)}}}\xrightarrow{p}\frac{1}{1+K^{(s)}\dfrac{c_0^2}{c_1^2}\dfrac{p_1^{(s)}(1-p_1^{(s)})}{p_0^{(s)}(1-p_0^{(s)})}} \tag{S.17}$$

Similarly, we can derive

$$\sqrt{\frac{c_0^2/M_0^{(s)}}{c_1^2/M_1^{(s)}+c_0^2/M_0^{(s)}}}\xrightarrow{p}\frac{1}{1+\dfrac{1}{K^{(s)}}\dfrac{c_1^2}{c_0^2}\dfrac{p_0^{(s)}(1-p_0^{(s)})}{p_1^{(s)}(1-p_1^{(s)})}} \tag{S.18}$$

We further explore the term $S_1^{(s)}/\sqrt{1/M_1^{(s)}}$ in Equation (S.13).

$$\frac{S_1^{(s)}}{\sqrt{1/M_1^{(s)}}}=\sqrt{n_1^{(s)}}\sqrt{\widehat{p}_1^{(s)}(1-\widehat{p}_1^{(s)})}\log\left(\frac{\widehat{p}_1^{(s)}}{1-\widehat{p}_1^{(s)}}\right) \tag{S.19}$$

We define $f(x)=\sqrt{x(1-x)}\log(x/(1-x))$. Then, $S_1^{(s)}/\sqrt{1/M_1^{(s)}}=\sqrt{n_1^{(s)}}f(\widehat{p}_1^{(s)})$. By (S.15) and the Delta method, we have

$$\sqrt{n_1^{(s)}}(f(\widehat{p}_1^{(s)})-f(p_1^{(s)}))\xrightarrow{d}N(0,(f'(p_1^{(s)}))^2p_1(1-p_1^{(s)})) \tag{S.20}$$

Let $Y_1^{(s)}$ be a random variable that follows $N(0,(f'(p_1^{(s)}))^2p_1(1-p_1^{(s)}))$, then we have $\sqrt{n_1^{(s)}}f(\widehat{p}_1^{(s)})\approx\sqrt{n_1^{(s)}}f(p_1^{(s)})+Y_1^{(s)}$. Similarly, we can obtain

$$S_0^{(s)}/\sqrt{1/M_0^{(s)}}=\sqrt{n_0^{(s)}}f(\widehat{p}_0^{(s)})\approx\sqrt{n_0^{(s)}}f(p_0^{(s)})+Y_0^{(s)} \tag{S.21}$$

where $Y_0^{(s)} \sim N(0, (f'(p_0^{(s)}))^2 p_0(1-p_0^{(s)}))$. Through Slutsky's theorem, we have

$$
\begin{aligned}
\tilde{t}^{(s)} &= \frac{c_1 S_1 + c_0 S_0}{\sqrt{c_1^2/M_1^{(s)} + c_0^2/M_0^{(s)}}} \\
&\approx \left(\sqrt{n_1^{(s)}} f(p_1^{(s)}) + Y_1^{(s)}\right) \sqrt{\frac{1}{1 + K^{(s)} \frac{c_0^2}{c_1^2} \frac{p_1^{(s)}(1-p_1^{(s)})}{p_0^{(s)}(1-p_0^{(s)})}}} + \left(\sqrt{n_0^{(s)}} f(p_0^{(s)}) + Y_0^{(s)}\right) \sqrt{\frac{1}{1 + \frac{1}{K^{(s)}} \frac{c_1^2}{c_0^2} \frac{p_0^{(s)}(1-p_0^{(s)})}{p_1^{(s)}(1-p_1^{(s)})}}}
\end{aligned} \tag{S.22}
$$

Given $p_0^{(s)}$ and $p_1^{(s)}$, the independence between the two groups (the disease group and non-disease group) naturally holds, thus, $Y_1^{(s)} \perp Y_0^{(s)}$. $\tilde{t}^{(s)}$ is a linear combination of two independent normally distributed variables, $Y_0^{(s)}$ and $Y_1^{(s)}$; therefore, it asymptotically follows a normal distribution. We can obtain the asymptotic expectation and variance for $\tilde{t}^{(s)}$, which are

$$
\begin{aligned}
E\left(\tilde{t}^{(s)} \mid p_1^{(s)}, p_0^{(s)}\right) &\approx \sqrt{n_1^{(s)}} f(p_1^{(s)}) \sqrt{\frac{1}{1 + K^{(s)} \frac{c_0^2}{c_1^2} \frac{p_1^{(s)}(1-p_1^{(s)})}{p_0^{(s)}(1-p_0^{(s)})}}} + \sqrt{n_0^{(s)}} f(p_0^{(s)}) \sqrt{\frac{1}{1 + \frac{1}{K^{(s)}} \frac{c_1^2}{c_0^2} \frac{p_0^{(s)}(1-p_0^{(s)})}{p_1^{(s)}(1-p_1^{(s)})}}} \\
\mathrm{Var}\left(\tilde{t}^{(s)} \mid p_1^{(s)}, p_0^{(s)}\right) &\approx (f'(p_1^{(s)}))^2 p_1(1-p_1^{(s)}) \frac{1}{1 + K^{(s)} \frac{c_0^2}{c_1^2} \frac{p_1^{(s)}(1-p_1^{(s)})}{p_0^{(s)}(1-p_0^{(s)})}} + \\
&\quad (f'(p_0^{(s)}))^2 p_0(1-p_0^{(s)}) \frac{1}{1 + \frac{1}{K^{(s)}} \frac{c_1^2}{c_0^2} \frac{p_0^{(s)}(1-p_0^{(s)})}{p_1^{(s)}(1-p_1^{(s)})}}
\end{aligned} \tag{S.23}
$$

We denote the approximate expectation and variance of $\tilde{t}^{(s)}$ given $p_1^{(s)}$ and $p_0^{(s)}$ by $S_0(p_1^{(s)}, p_0^{(s)})$ and $S_1(p_1^{(s)}, p_0^{(s)})$. Suppose the set of parameters for the bivariate binomial model is denoted by $\Theta = (\theta, \alpha, \sigma_\theta, \sigma_\alpha, \beta)$. We can estimate the unconditional expectation and variance in the following manner:

$$
\begin{aligned}
E\left(\tilde{t}^{(s)}\right) &= E_{p_1^{(s)}, p_0^{(s)}} E\left(t^{(s)} \mid p_1^{(s)}, p_0^{(s)}\right) \approx \int_{-\infty}^{\infty} \int_{-\infty}^{\infty} S_0(p_1^{(s)}, p_0^{(s)}) f(p_1^{(s)}, p_0^{(s)} \mid \Theta) \, \mathrm{d}p_1^{(s)} \, \mathrm{d}p_0^{(s)} \\
\mathrm{Var}\left(\tilde{t}^{(s)}\right) &= \mathrm{Var}_{p_1^{(s)}, p_0^{(s)}} \left(E(t^{(s)} \mid p_1^{(s)}, p_0^{(s)})\right) + E_{p_1^{(s)}, p_0^{(s)}} \left(\mathrm{Var}(t^{(s)} \mid p_1^{(s)}, p_0^{(s)})\right) \\
&\approx \int_{-\infty}^{\infty} \int_{-\infty}^{\infty} S_0^2(p_1^{(s)}, p_0^{(s)}) f(p_1^{(s)}, p_0^{(s)} \mid \Theta) \, \mathrm{d}p_1^{(s)} \, \mathrm{d}p_0^{(s)} \\
&\quad - \left(\int_{-\infty}^{\infty} \int_{-\infty}^{\infty} S_0(p_1^{(s)}, p_0^{(s)}) f(p_1^{(s)}, p_0^{(s)} \mid \Theta) \, \mathrm{d}p_1^{(s)} \, \mathrm{d}p_0^{(s)}\right)^2 \\
&\quad + \int_{-\infty}^{\infty} \int_{-\infty}^{\infty} S_1(p_1^{(s)}, p_0^{(s)}) f(p_1^{(s)}, p_0^{(s)} \mid \Theta) \, \mathrm{d}p_1^{(s)} \, \mathrm{d}p_0^{(s)},
\end{aligned} \tag{S.24}
$$

where $f(p_1^{(s)}, p_0^{(s)} \mid \Theta)$ means the joint p.d.f. of $p_1^{(s)}, p_0^{(s)}$ given parameters Θ.

Finally, we can obtain the approximation for the marginal selection probability as

$$
\mathrm{P}\left(\text{select} \mid n_1^{(s)}, n_0^{(s)}\right) \approx E_{t \sim N(E(t^{(s)}), \mathrm{Var}(t^{(s)}))} H(\gamma_0 + \gamma_1 t). \tag{S.25}
$$

If we consider the probit function for $H(\cdot)$, the marginal selection probability reduces to

$$
\mathrm{P}\left(\text{select} \mid n_1^{(s)}, n_0^{(s)}\right) \approx \Phi\left(\frac{\gamma_0 + \gamma_1 E(t^{(s)})}{\sqrt{1 + \gamma_1^2 \mathrm{Var}(t^{(s)})}}\right). \tag{S.26}
$$

Web Appendix D | APPLICATIONS ON IVD DATA

We illustrated our sensitivity analysis method with the data in Example 2 of Safdar et al.[23]. Hattori and Zhou[22] and Zhou et al.[19] used this dataset for their illustration. The meta-analysis investigated the effect of semi-quantitative and quantitative catheter segment culture tests on diagnosing intravascular device (IVD) related bloodstream infection with 33 individual studies. The data is presented in Table 2. Of the 33 independent studies, nine studies had zero frequency of false positive (FP) or false negative (FN) and most of the other studies had very low frequencies in the above two cells. In the original paper, Safdar et al.[23] estimated the SAUC as 0.934 (95% CI: [0.902, 0.951]), suggesting the high diagnostic capacity of the semi-quantitative and quantitative catheter segment culture tests in diagnosing IVD. The overall sensitivity and specificity were estimated as 0.862 (95% CI: [0.829, 0.894]) and 0.871 (95% CI: [0.838, 0.903]), respectively. We re-analyzed this meta-analysis data, whose original analysis did not take into account of PB and the sparsity of data. We conducted sensitivity analysis setting $p = 0.2, 0.4, 0.6, 0.8, 1$ with three specified selective mechanisms: $(c_0, c_1) = (1/\sqrt{2}, 1/\sqrt{2})$, $(c_0, c_1) = (1, 0)$, and $(c_0, c_1) = (0, 1)$. Note that $p = 1$ implies the original SROC curve analysis without adjusting for selective publication. In our sensitivity analysis, the link function $G(\cdot)$ is also chosen as the standard logistic function.

WEB TABLE 2: Meta-analysis of IVD dataset

Study	TP	FN	FP	TN
1	12	0	29	289
2	10	2	14	72
3	17	1	36	85
4	13	0	18	67
5	4	0	21	225
6	15	2	122	403
7	45	5	28	34
8	18	4	69	133
9	5	0	11	34
10	8	9	15	96
11	5	0	7	63
12	11	2	122	610
13	5	1	6	145
14	7	5	25	342
15	10	1	93	296
16	5	5	41	271
17	5	0	15	53
18	55	13	19	913
19	6	2	12	30
20	42	26	19	913
21	5	3	5	37
22	13	0	11	125
23	20	0	24	287
24	7	6	13	72
25	48	2	15	47
26	11	1	14	72
27	15	5	32	170
28	68	13	5	11
29	13	1	5	72
30	8	3	66	323
31	13	1	98	293
32	14	1	0	155

33	8	2	4	60

We showed the estimated SAUCs with the corresponding 95% CI and the estimated SROC curves under each given selection probability p in Web Figure 1. In the upper panel of Web Figure 1, we depicted the estimated SROC curves and traced the SOPs under various p. As shown in panel (A) of Web Figure 1, the plots of the SOPs suggested a selective publication process under which studies around the lower right part of the SROC curve were less likely published. The change of the SROC curve and SAUC suggested that the result was robust against this kind of selective publication mechanism. Tracing the SOPs in the panel (B) to (C) of Web Figure 1, one can understand that the selection function with $(c_0, c_1) = (0, 1)$ and $= (1, 0)$ modeled different publication mechanisms and the figures indicated that impacts by these selective publication processes would be minor. We showed the estimated SAUC with varying p in panels (D) to (F) of Web Figure 1. The SAUC was 0.931 (95% CI: [0.906, 0.950]) without accounting for selective publication ($p = 1$). With all assumed p, the lower bound of the 95% confidence interval for SAUC was larger than 0.5 under all three selective mechanisms, suggesting that the test was useful to diagnose IVD since SAUC=0.5 indicates that the diagnostic test result is a random guess. Of the three selective mechanisms, the estimated SAUCs under $c_0 = c_1$ showed a larger difference between $p = 1$ and $p = 0.2$ compared with the other two selective mechanisms, suggesting that considerable PB would exist if lnDOR were affecting the selection. Our proposal also suggested the estimated SAUC with the bivariate binomial model wouldn't change much between $p = 1$ and $p = 0.4$ under the selection mechanisms $(c_0, c_1) = (1, 0)$ and $(c_0, c_1) = (0, 1)$. When assuming $c_0 = c_1$, the SAUC under $p = 1$ would be 0.2 lower than that under $p = 1$, showing PB largely affected the estimate of SAUC.

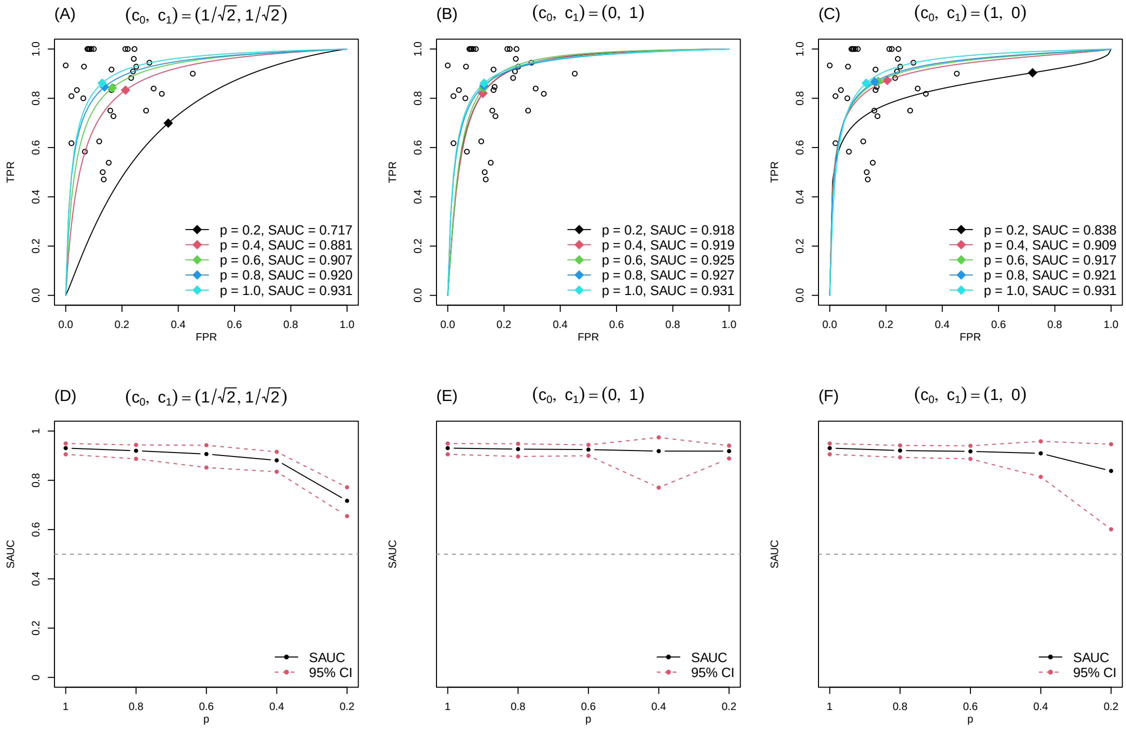

WEB FIGURE 1 The estimated SROC curves and SAUC under three selective publication mechanisms in IVD example (our proposal).

We also conducted the sensitivity analysis based on the bivariate normal model by Zhou et al.[19] for comparisons. The estimated SROC curves were given in panels (A) to (C) of Web Figure 2 given different selective mechanisms under $p = 0.2, 0.4, 0.6, 0.8, 1$; the variation of SAUC was shown in panels (D) to (F) of Web Figure 2. Similar to the results for CD64 datasets, the bivariate normal model obtained lower estimates compared with the bivariate binomial model in most cases. In contrast, the bivariate normal model suggested the estimated SAUC gradually decreased with p declining no matter what the selective mechanism was.

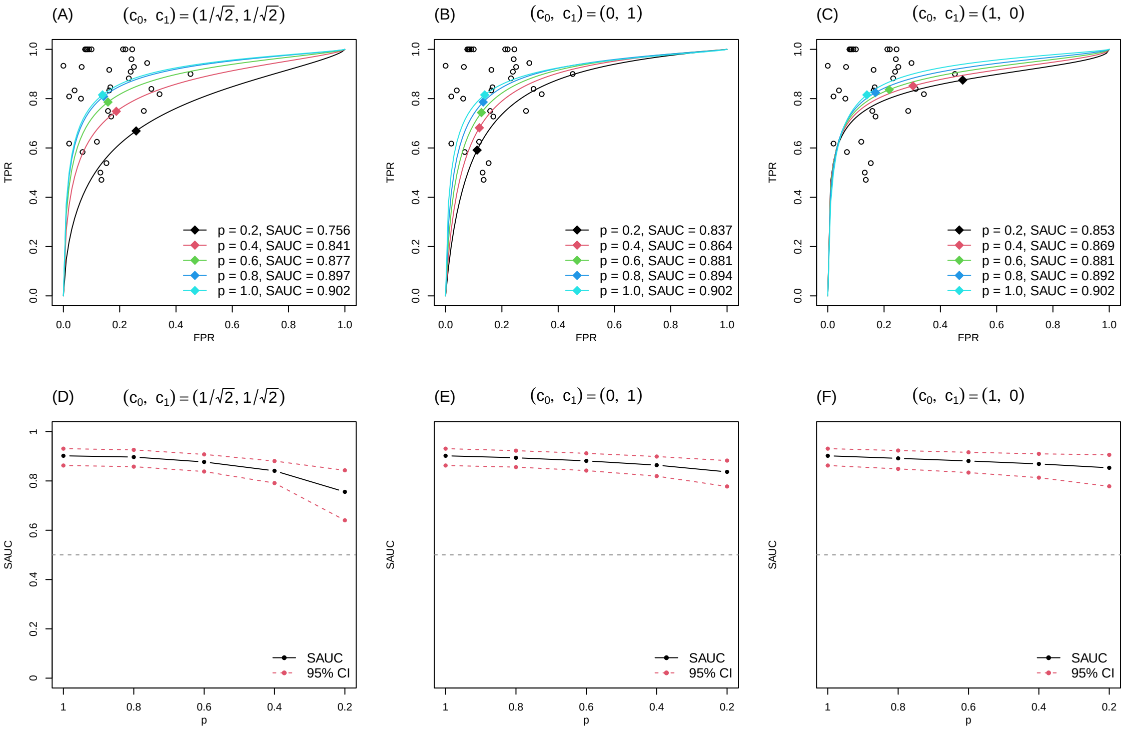

WEB FIGURE 2 The estimated SROC curves and SAUC under three selective publication mechanisms in IVD example with the bivariate normal model by Zhou et al. (2023).

Web Appendix E | APPLICATIONS ON CERVICAL DATA

We conducted another real-data analysis using the meta-analysis by Scheidler et al.[24], referred to as the Cervical data. This meta-analysis investigated the utility of lymphangiography (LAG), computed tomography (CT), and magnetic resonance (MR) imaging for diagnosing lymph node metastasis in patients with cervical cancer with 44 individual studies, as presented in Web Table 3. Among the 44 studies, 4 studies had zero false positives or false negatives. The SAUC was estimated as 0.849 (95% CI: [0.798, 0.889]) without considering PB, suggesting the high efficiency of LAG, CT, and MR imaging in diagnosing cervical cancer. The sensitivity and specificity were estimated as 0.577 (95% CI: [0.440, 0.714]) and 0.911 (95% CI: [0.876, 0.947]), respectively. We conducted sensitivity analysis setting $p = 0.2, 0.4, 0.6, 0.8, 1$ with three specified selective mechanisms: $(c_0, c_1) = (1/\sqrt{2}, 1/\sqrt{2})$, $(c_0, c_1) = (1, 0)$, and $(c_0, c_1) = (0, 1)$. Note that $p = 1$ implies the original SROC curve analysis without accounting for selective publication. Within the sensitivity analysis, the link function $G(\cdot)$ is chosen as the standard logistic function.

WEB TABLE 3: Meta-analysis of Cervical data.

Study No.	Author	Year	Method	TP	FP	FN	TN
1	Grumbine	1981	LAG	0	1	6	17
2	Walsh	1981	LAG	12	3	3	7
3	Brenner	1982	LAG	4	1	2	13
4	Villasanta	1983	LAG	10	4	3	25
5	vanEngelsho	1984	LAG	3	1	4	12
6	Bandy	1985	LAG	9	3	3	29
7	Vas	1985	LAG	20	4	8	31

8	King	1986	LAG	17	5	7	21
9	Feigen	1987	LAG	2	0	9	32
10	Camilien	1988	LAG	3	1	9	38
11	Janus	1989	LAG	1	1	2	18
12	Matsukuma	1989	LAG	5	2	2	61
13	Heller	1990	LAG	21	8	40	184
14	Kim	1990	LAG	4	3	9	42
15	Ho	1992	LAG	0	0	5	15
16	Kim	1993	LAG	7	11	22	158
17	Subak	1995	LAG	3	3	2	29
18	Kindermann	1970	CT	19	1	10	81
19	Lecart	1971	CT	8	9	2	13
20	Piver	1971	CT	41	1	12	49
21	Piver	1973	CT	5	1	2	18
22	Kolbenstvedt	1975	CT	45	58	32	165
23	LemanJr	1975	CT	8	6	2	32
24	Brown	1979	CT	5	8	1	7
25	Lagasse	1979	CT	15	17	11	52
26	Kjorstad	1980	CT	16	11	8	24
27	Ashraf	1982	CT	4	8	2	25
28	deMuylder	1984	CT	8	12	10	70
29	Smales	1986	CT	10	4	4	55
30	Feigen	1987	CT	2	5	6	23
31	Swart	1989	CT	7	10	7	30
32	Heller	1990	CT	44	50	12	135
33	Lafianza	1990	CT	8	3	1	37
34	Stellato	1992	CT	4	3	0	14
35	Hricak	1988	MR	9	2	2	44
36	Greco	1989	MR	3	6	5	32
37	Janus	1989	MR	3	2	1	16
38	Kim	1990	MR	3	1	12	44
39	Ho	1992	MR	0	0	5	15
40	Kim	1993	MR	7	2	22	167
41	Hawnaur	1994	MR	12	4	4	29
42	Kim	1994	MR	23	5	14	230
43	Subak	1995	MR	8	5	5	53
44	Heuck	1997	MR	16	2	2	22

We showed the estimated SAUCs with the corresponding 95% CI and the estimated SROC curves under each given selection probability p in Web Figure 3. In the upper panel of Web Figure 3, we depicted the estimated SROC curves and traced the SOPs under various p. As shown in panel (A) of Web Figure 3, the plots of the SOPs suggested a selective publication process under which studies around the lower right part of the SROC curve were less likely published. The change of the SROC curve and SAUC suggested that the result was robust against this kind of selective publication mechanism. Tracing the SOPs in the panel (B) to (C) of Web Figure 3, one can understand that the selection function with $(c_0, c_1) = (0, 1)$ and $= (1, 0)$ modeled different publication mechanisms and the figures indicated that impacts by these selective publication processes would be minor. We showed the estimated SAUC with varying p in panels (D) to (F) of Web Figure 3. The SAUC was 0.849 (95% CI: [0.798, 0.889]) without accounting for selective publication ($p = 1$). With all assumed p, the lower bound for SAUC was larger than 0.5 under all three selective mechanisms, suggesting that the test was useful to diagnose cervical. Of the three selective mechanisms, the estimated SAUCs under $c_0 = c_1$ showed a larger difference between $p = 1$ and $p = 0.2$ compared with the other two selective mechanisms, suggesting that considerable PB would exist if both the sensitivity and specificity were affecting the selection. Our proposal also suggested that the estimated SAUC with the bivariate binomial model scarcely changed between $p = 1$ and

$p = 0.4$ under the selection mechanisms $(c_0, c_1) = (1, 0)$ and $(c_0, c_1) = (0, 1)$. When assuming $c_0 = c_1$, the SAUC under $p = 1$ would be 0.1 lower than that under $p = 1$, showing PB largely affected the estimate of SAUC assuming the selection mechanism when $c_0 = c_1$.

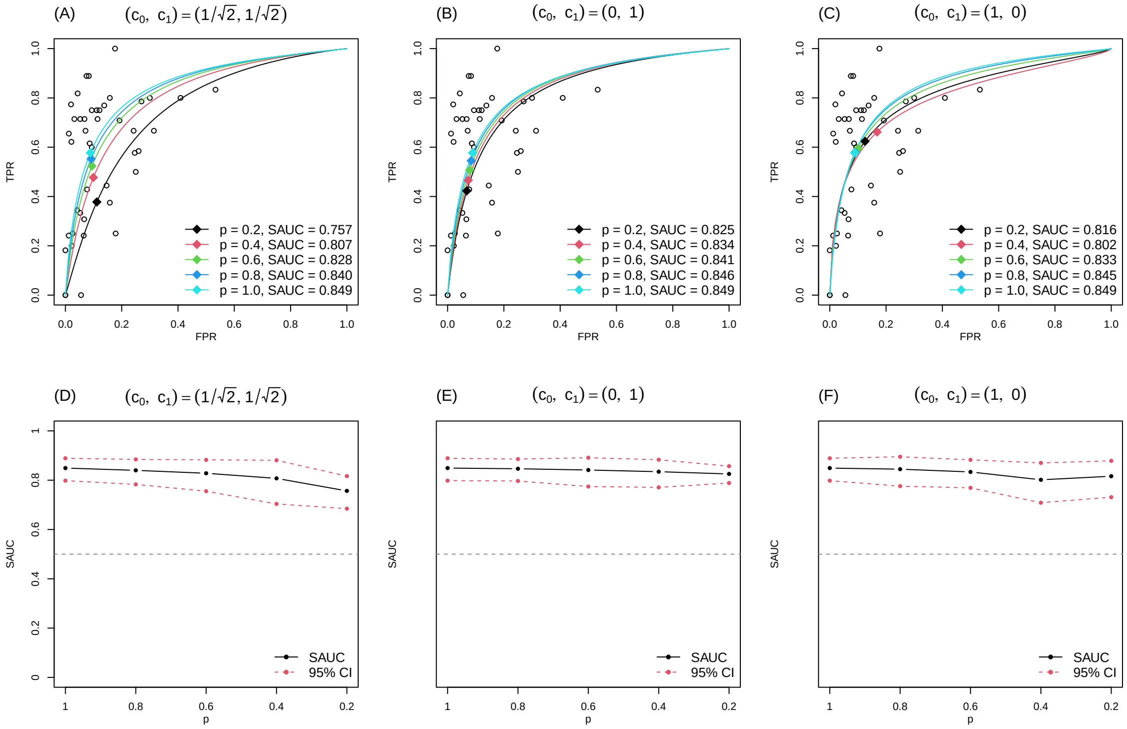

WEB FIGURE 3 The estimated SROC curves and SAUC under three selective publication mechanisms in the cervical example (our proposal).

We also conducted the sensitivity analysis based on the bivariate normal model by Zhou et al.[19] for comparisons. The estimated SROC curves were given in panels (A) to (C) of Web Figure 4 given different selective mechanisms under $p = 0.2, 0.4, 0.6, 0.8, 1$; the variation of SAUC was shown in panels (D) to (F) of Web Figure 4. Like the previous two examples, the bivariate normal model obtained lower estimates compared with the bivariate binomial model in most cases. Under the selective mechanism $(c_0, c_1) = (1, 0)$, the estimated SAUC did not show any changes with p varying from 0.2 to 1, and the estimated confidence interval was unstable.

WEB TABLE 4 Scenarios for simulation studies

Experiment	SAUC	Sensitivity	Specificity	θ	α	σ_θ	σ_α	γ_1
1	0.832	0.9	0.5	1.184	2.368	0.6	1.2	1.5
2	0.798	0.5	0.9	-1.019	2.038	0.6	1.2	1.5
3	0.869	0.8	0.8	0.104	2.78	0.6	1.2	1.5
4	0.832	0.9	0.5	1.184	2.368	1.2	0.6	1.5
5	0.798	0.5	0.9	-1.019	2.038	1.2	0.6	1.5
6	0.869	0.8	0.8	0.104	2.78	1.2	0.6	1.5

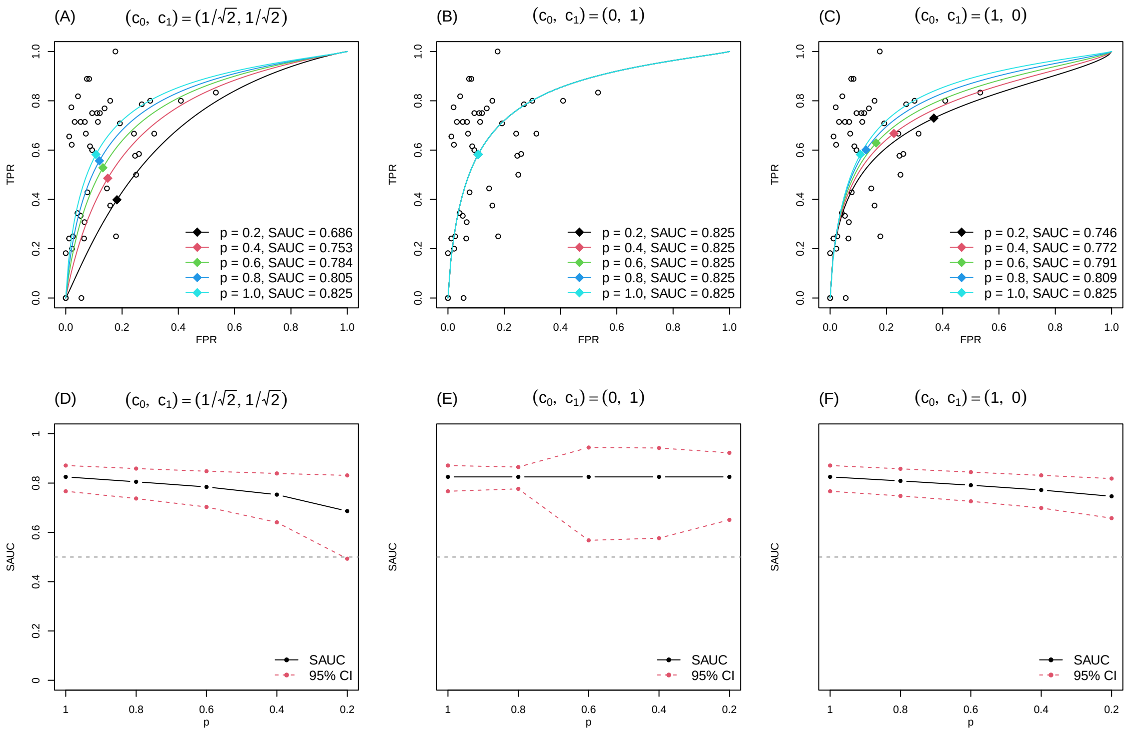

WEB FIGURE 4 The estimated SROC curves and SAUC under three selective publication mechanisms in cervical example with the bivariate normal model by Zhou et al. (2023).

Web Appendix F | SCENARIOS FOR SIMULATION STUDIES

We summarized the parameters for 6 scenarios of the simulation studies in Web Table 4.

Web Appendix G | RESULTS FOR SIMULATION STUDIES

In this appendix, we show the complete results of the simulation studies, which only a part of have been reported in Section 5 in our main body.

In addition to the selective mechanism as $(c_0, c_1) = (1/\sqrt{2}, 1/\sqrt{2})$, whose core results are presented in Section 5, we conducted the sensitivity analysis under the rest of two selective mechanisms as $(c_0, c_1) = (0, 1)$ and $(c_0, c_1) = (1, 0)$. The procedures for generating the published and unpublished studies of meta-analysis remained the same with the simulation studies under $(c_0, c_1) = (1/\sqrt{2}, 1/\sqrt{2})$. The selective mechanism $(c_0, c_1) = (0, 1)$ suggested the publication process was determined by the sensitivity; the selective mechanism $(c_0, c_1) = (1, 0)$ suggested the publication process was determined by the specificity.

WEB TABLE 5 Summary of the sparsity of the simulated datasets under $(c_0, c_1) = (1/\sqrt{2}, 1/\sqrt{2})$ given $p \approx 0.7$. (Full indicates published and unpublished studies; Published indicates published studies)

Experiment	Rate(%)	S=15		S=25		S=50		S=100	
		Full	Published	Full	Published	Full	Published	Full	Published
1	Zero entries	21.0	19.7	20.9	19.7	20.8	19.3	21.5	20.2
	No-more-than-3-entries	80.5	75.4	80.4	74.9	80.8	75.2	81.0	75.8
	No-more-than-5-entries	93.4	90.1	93.3	90.0	93.8	90.2	93.5	90.2
2	Zero entries	0.6	0.9	0.6	0.9	0.7	1.0	0.8	1.1
	No-more-than-3-entries	15.0	18.3	14.9	18.6	15.1	18.4	15.7	18.4
	No-more-than-5-entries	38.4	43.1	38.2	43.5	38.4	43.2	38.8	42.9
3	Zero entries	5.4	6.7	5.5	6.9	5.3	6.7	5.0	6.5
	No-more-than-3-entries	50.0	47.8	49.4	46.9	49.5	47.0	48.2	46.0
	No-more-than-5-entries	73.6	70.7	73.3	70.2	73.5	70.3	72.9	70.2
4	Zero entries	15.5	23.2	15.2	23.3	15.5	23.3	14.9	22.7
	No-more-than-3-entries	63.6	70.5	63.2	70.7	63.6	70.9	64.2	71.3
	No-more-than-5-entries	80.2	84.7	80.3	84.9	80.7	85.1	81.6	85.8
5	Zero entries	1.4	3.6	1.3	3.7	1.4	3.6	1.3	4.1
	No-more-than-3-entries	21.4	30.5	21.5	30.4	21.1	30.1	21.5	30.9
	No-more-than-5-entries	45.9	54.4	45.9	54.1	44.9	53.5	45.3	54.0
6	Zero entries	4.1	10.7	4.0	11.1	3.4	10.8	3.3	10.9
	No-more-than-3-entries	40.0	50.7	40.7	51.5	39.2	50.7	40.5	51.6
	No-more-than-5-entries	64.4	71.8	64.5	72.2	63.7	71.6	64.0	71.7

We first conducted the sensitivity analysis under the true selective mechanism as $(c_0, c_1) = (1/\sqrt{2}, 1/\sqrt{2})$. We evaluated the sparsity of the simulated dataset in Web Table 5 and proved that the simulated dataset was sparse. We applied our proposed method to only published studies and compared our proposal with the sensitivity analysis proposed by Zhou et al.[19] based on the bivariate normal model, and the maximum likelihood estimation (MLE) obtained with only published studies based on the bivariate binomial model, which was the standard meta-analysis method without adjusting for PB. To make fair comparisons, we assumed the marginal selection probability p in both our proposed method and the sensitivity method by Zhou et al.[19] as 0.7, and the t-statistics in both methods were correctly specified with $(c_0, c_1) = (1/\sqrt{2}, 1/\sqrt{2})$. We showed the true parameter for sensitivities and specificities, the cutoff parameter and accuracy parameter θ and α for the bivariate binomial model, and SAUC in Web Table 6. The average for each parameter with the MLE with only the published studies had a non-ignorable discrepancy with the true value, indicating that PB was a considerable issue for this dataset. Both the proposed method and the method by Zhou et al.[19] successfully reduced biases. Our proposed method had only negligible and smaller biases than the method by Zhou et al.[19].

We then conducted the sensitivity analysis under $(c_0, c_1) = (0, 1)$. We evaluated the proportion of the studies with zero entries in the 2×2 table among all the studies (published and unpublished), as well as among the published studies, and the proportion of the studies with cell frequencies of no more than 3 and no more than 5 to show the sparsity of simulated datasets. Their averages over the 1000 simulated datasets are summarized in Web Table 7. It indicates that we successfully generated meta-analyses of sparsity.

We applied our proposed method to only published studies and compared our proposal with the sensitivity analysis proposed by Zhou et al.[19] based on the bivariate normal model, and the maximum likelihood estimation (MLE) obtained with only published studies based on the bivariate binomial model, which was the standard meta-analysis method without adjusting for PB. To make fair comparisons, we assumed the marginal selection probability p in both our proposed method and the sensitivity method by Zhou et al.[19] as 0.7, and the t-statistics in both methods were correctly specified with $(c_0, c_1) = (0, 1)$. We showed the true parameter for sensitivities and specificities, the cutoff parameter and accuracy parameter θ and α for the bivariate binomial model, and SAUC in Web Table 8. The average for each parameter with the MLE with only the published study had a non-ignorable discrepancy with the true value, indicating that PB was a considerable issue for this dataset. Both the proposed method and the method by Zhou et al.[19] successfully reduced biases. Our proposed method had only negligible and smaller biases than the method by Zhou et al.[19].

We finally conducted the sensitivity analysis under $(c_0, c_1) = (1, 0)$. The measures of sparsity were summarized in Web Table 9. It indicates that we successfully generated meta-analyses of sparsity.

WEB TABLE 6 Summary of the estimates under the true selection mechanism of $(c_0, c_1) = (1/\sqrt{2}, 1/\sqrt{2})$ given $p \approx 0.7$. (The estimates are summarized by mean (empirical standard error) over 1000 simulated meta-analyses; – for θ, α within the results for Zhou et al.[19] is because the θ, α is not available for the bivariate normal model)

Experiment		True	$S = 15$			$S = 25$			$S = 50$			$S = 100$		
			Our proposal	Bivariate binomial with published studies	Method of Zhou et al.[19]	Our proposal	Bivariate binomial with published studies	Method of Zhou et al.[19]	Our proposal	Bivariate binomial with published studies	Method of Zhou et al.[19]	Our proposal	Bivariate binomial with published studies	Method of Zhou et al.[19]
1	Sensitivity	0.900	0.919 (0.035)	0.910 (0.028)	0.866 (0.031)	0.923 (0.029)	0.910 (0.021)	0.864 (0.023)	0.930 (0.023)	0.911 (0.015)	0.864 (0.016)	0.936 (0.017)	0.912 (0.010)	0.864 (0.011)
	Specificity	0.500	0.550 (0.076)	0.580 (0.068)	0.555 (0.072)	0.553 (0.062)	0.583 (0.054)	0.557 (0.058)	0.561 (0.043)	0.587 (0.037)	0.561 (0.040)	0.564 (0.036)	0.585 (0.030)	0.560 (0.032)
	θ	1.184	1.185 (0.533)	1.391 (0.572)	-	1.078 (0.431)	1.322 (0.549)	-	0.975 (0.250)	1.231 (0.377)	-	0.942 (0.180)	1.155 (0.238)	-
	α	2.368	2.857 (0.866)	3.343 (0.979)	-	2.684 (0.691)	3.229 (0.957)	-	2.559 (0.415)	3.084 (0.648)	-	2.536 (0.279)	2.939 (0.403)	-
	SAUC	0.832	0.844 (0.058)	0.875 (0.044)	0.848 (0.043)	0.839 (0.047)	0.874 (0.035)	0.848 (0.033)	0.837 (0.034)	0.876 (0.025)	0.851 (0.024)	0.836 (0.025)	0.875 (0.017)	0.851 (0.017)
2	Sensitivity	0.500	0.520 (0.076)	0.571 (0.064)	0.527 (0.068)	0.518 (0.061)	0.576 (0.049)	0.531 (0.054)	0.513 (0.043)	0.577 (0.036)	0.530 (0.039)	0.518 (0.029)	0.584 (0.024)	0.537 (0.026)
	Specificity	0.900	0.903 (0.030)	0.919 (0.022)	0.900 (0.028)	0.901 (0.024)	0.920 (0.017)	0.900 (0.023)	0.902 (0.017)	0.922 (0.011)	0.902 (0.015)	0.901 (0.013)	0.922 (0.007)	0.902 (0.012)
	θ	-1.019	-0.986 (0.473)	-0.861 (0.373)	-	-0.970 (0.387)	-0.854 (0.287)	-	-1.006 (0.269)	-0.891 (0.189)	-	-0.989 (0.208)	-0.882 (0.133)	-
	α	2.038	2.219 (0.763)	2.450 (0.614)	-	2.162 (0.584)	2.470 (0.439)	-	2.146 (0.422)	2.517 (0.313)	-	2.148 (0.317)	2.549 (0.237)	-
	SAUC	0.798	0.791 (0.085)	0.817 (0.074)	0.762 (0.094)	0.794 (0.066)	0.827 (0.052)	0.768 (0.070)	0.801 (0.050)	0.838 (0.037)	0.780 (0.051)	0.805 (0.039)	0.844 (0.027)	0.783 (0.039)
3	Sensitivity	0.800	0.819 (0.060)	0.816 (0.042)	0.774 (0.044)	0.818 (0.050)	0.816 (0.031)	0.773 (0.032)	0.815 (0.041)	0.816 (0.021)	0.772 (0.022)	0.809 (0.033)	0.814 (0.015)	0.770 (0.016)
	Specificity	0.800	0.816 (0.048)	0.843 (0.037)	0.826 (0.042)	0.819 (0.037)	0.848 (0.027)	0.833 (0.031)	0.818 (0.027)	0.848 (0.020)	0.833 (0.022)	0.819 (0.019)	0.849 (0.014)	0.834 (0.015)
	θ	0.104	0.091 (0.628)	0.239 (0.583)	-	-0.015 (0.507)	0.157 (0.444)	-	-0.115 (0.340)	0.084 (0.290)	-	-0.159 (0.230)	0.050 (0.180)	-
	α	2.780	3.281 (0.622)	3.407 (0.536)	-	3.206 (0.528)	3.338 (0.406)	-	3.092 (0.410)	3.263 (0.250)	-	3.009 (0.316)	3.221 (0.143)	-
	SAUC	0.869	0.878 (0.041)	0.888 (0.031)	0.846 (0.043)	0.881 (0.034)	0.893 (0.024)	0.850 (0.036)	0.882 (0.025)	0.897 (0.015)	0.856 (0.025)	0.880 (0.019)	0.897 (0.010)	0.857 (0.019)
4	Sensitivity	0.900	0.898 (0.050)	0.865 (0.048)	0.823 (0.046)	0.905 (0.043)	0.865 (0.039)	0.823 (0.036)	0.918 (0.029)	0.868 (0.028)	0.825 (0.026)	0.923 (0.021)	0.868 (0.020)	0.825 (0.019)
	Specificity	0.500	0.557 (0.095)	0.621 (0.091)	0.592 (0.095)	0.553 (0.074)	0.624 (0.070)	0.593 (0.075)	0.553 (0.056)	0.626 (0.053)	0.595 (0.056)	0.551 (0.045)	0.625 (0.041)	0.593 (0.044)
	θ	1.184	0.976 (0.472)	0.794 (0.450)	-	0.978 (0.357)	0.762 (0.314)	-	0.970 (0.253)	0.750 (0.224)	-	0.976 (0.197)	0.753 (0.172)	-
	α	2.368	2.489 (0.477)	2.561 (0.487)	-	2.440 (0.315)	2.506 (0.269)	-	2.438 (0.202)	2.496 (0.176)	-	2.427 (0.129)	2.484 (0.118)	-
	SAUC	0.832	0.830 (0.038)	0.840 (0.031)	0.812 (0.033)	0.831 (0.028)	0.841 (0.022)	0.811 (0.023)	0.833 (0.020)	0.843 (0.015)	0.811 (0.016)	0.833 (0.013)	0.843 (0.010)	0.811 (0.011)
5	Sensitivity	0.500	0.502 (0.107)	0.540 (0.079)	0.523 (0.074)	0.494 (0.091)	0.542 (0.060)	0.525 (0.059)	0.491 (0.065)	0.543 (0.041)	0.529 (0.040)	0.498 (0.049)	0.549 (0.027)	0.536 (0.026)
	Specificity	0.900	0.899 (0.046)	0.906 (0.031)	0.887 (0.037)	0.900 (0.040)	0.906 (0.025)	0.886 (0.030)	0.903 (0.027)	0.907 (0.016)	0.887 (0.020)	0.902 (0.020)	0.907 (0.009)	0.886 (0.013)
	θ	-1.019	-1.042 (0.518)	-0.927 (0.339)	-	-1.079 (0.452)	-0.927 (0.262)	-	-1.084 (0.332)	-0.915 (0.172)	-	-1.053 (0.264)	-0.892 (0.110)	-
	α	2.038	2.132 (0.484)	2.220 (0.367)	-	2.144 (0.368)	2.236 (0.263)	-	2.115 (0.284)	2.224 (0.187)	-	2.107 (0.218)	2.231 (0.131)	-
	SAUC	0.798	0.796 (0.057)	0.809 (0.043)	0.773 (0.055)	0.803 (0.043)	0.814 (0.030)	0.776 (0.039)	0.802 (0.034)	0.814 (0.022)	0.774 (0.028)	0.803 (0.026)	0.816 (0.015)	0.774 (0.021)
6	Sensitivity	0.800	0.779 (0.086)	0.749 (0.063)	0.717 (0.061)	0.795 (0.068)	0.751 (0.049)	0.719 (0.045)	0.800 (0.053)	0.748 (0.034)	0.716 (0.031)	0.809 (0.039)	0.749 (0.026)	0.717 (0.025)
	Specificity	0.800	0.816 (0.068)	0.855 (0.049)	0.837 (0.054)	0.815 (0.053)	0.859 (0.035)	0.842 (0.039)	0.816 (0.037)	0.863 (0.023)	0.846 (0.026)	0.815 (0.030)	0.865 (0.018)	0.848 (0.019)
	θ	0.104	-0.085 (0.530)	-0.215 (0.390)	-	-0.051 (0.406)	-0.220 (0.281)	-	-0.074 (0.309)	-0.254 (0.194)	-	-0.047 (0.234)	-0.256 (0.157)	-
	α	2.780	2.930 (0.370)	2.914 (0.275)	-	2.947 (0.320)	2.915 (0.221)	-	2.947 (0.262)	2.902 (0.158)	-	2.961 (0.162)	2.910 (0.099)	-
	SAUC	0.869	0.870 (0.031)	0.873 (0.023)	0.840 (0.033)	0.875 (0.027)	0.875 (0.019)	0.843 (0.025)	0.878 (0.021)	0.877 (0.013)	0.844 (0.017)	0.882 (0.012)	0.878 (0.008)	0.845 (0.011)

WEB TABLE 7 Summary of the sparsity of simulated datasets under $(c_0, c_1) = (0, 1)$ given $p \approx 0.7$. Full indicates published and unpublished studies; Published indicates published studies.

Experiment	Rate(%)	S=15		S=25		S=50		S=100	
		Full	Published	Full	Published	Full	Published	Full	Published
1	Zero entries	20.2	20.0	19.9	19.7	19.8	19.6	19.7	19.6
	No-more-than-3-entries	75.5	75.6	75.6	75.5	74.9	75.2	75.7	76.0
	No-more-than-5-entries	90.2	90.3	90.3	90.1	89.9	90.0	90.6	90.5
2	Zero entries	0.6	0.7	0.7	0.8	0.8	0.9	0.9	1.0
	No-more-than-3-entries	16.7	18.5	16.5	18.4	16.3	18.2	16.4	18.3
	No-more-than-5-entries	40.6	43.1	40.7	43.1	39.8	42.4	40.4	42.7
3	Zero entries	6.6	6.4	7.1	7.0	6.5	6.6	6.7	6.8
	No-more-than-3-entries	46.0	46.1	47.1	47.1	47.0	47.0	46.6	46.3
	No-more-than-5-entries	69.5	69.5	70.8	70.7	70.4	70.2	69.6	69.4
4	Zero entries	12.8	23.2	12.0	22.6	11.8	23.1	11.6	23.2
	No-more-than-3-entries	59.1	70.3	59.4	70.5	59.5	70.6	59.4	70.8
	No-more-than-5-entries	78.7	84.9	78.4	84.7	78.9	85.1	79.1	85.2
5	Zero entries	1.5	3.8	1.2	3.7	1.2	3.6	1.5	4.1
	No-more-than-3-entries	20.6	30.2	20.0	30.4	19.9	30.1	20.3	30.9
	No-more-than-5-entries	45.7	54.1	45.1	54.2	44.7	53.7	45.1	54.0
6	Zero entries	4.0	10.6	3.4	10.2	3.5	10.6	3.8	11.0
	No-more-than-3-entries	37.1	50.7	35.8	50.3	35.9	50.8	35.8	50.6
	No-more-than-5-entries	61.6	71.3	61.1	71.3	60.7	71.2	61.2	71.3

We applied our proposed method to only published studies and compared our proposal with the sensitivity analysis proposed by Zhou et al.[19] based on the bivariate normal model, and the maximum likelihood estimation (MLE) obtained with only published studies based on the bivariate binomial model. To make fair comparisons, we assumed the marginal selection probability p in both our proposed method and the sensitivity method by Zhou et al.[19] as 0.7, and the t-statistics in both methods were correctly specified with $(c_0, c_1) = (1, 0)$. The average mean estimates and the sample standard deviation among the 1000 simulated meta-analyses for the parameters and SAUC were shown in Web Table 10. The results suggested our proposed method had smaller biases than Zhou et al.[19] and MLE with only published studies.

The simulation studies under all three selective mechanisms $(c_0, c_1) = (1/\sqrt{2}, 1/\sqrt{2}), (1, 0), (0, 1)$ substantiated the good performance of our proposal in reducing PB.

Web Appendix H | SIMULATION STUDIES UNDER THE MARGINAL SELECTION PROBABILITY SET AS 0.5

To examine the robustness of our proposed method with different marginal selection probability p, we conducted additional simulation studies, following the same set-ups as Section 4 but changing the marginal selection probability p from 0.7 to 0.5 and applying the true selective mechanism as $(c_0, c_1) = (1/\sqrt{2}, 1/\sqrt{2})$. Specifically, we considered the six scenarios as in Web Appendix F. Under each scenario, we considered simulating small-scale, medium-scale, and relatively large-scale meta-analyses with $\tilde{S}$ ($\tilde{S} = 15, 25, 50$) published and unpublished studies and we generated 1000 independent meta-analyses with $\tilde{S}$ studies. We considered the selection function based on the t-statistics for the lnDOR, which corresponded to $(c_0, c_1) = (1/\sqrt{2}, 1/\sqrt{2})$ given the fixed marginal publication probability p as 0.5, suggesting around 50% of studies would be unpublished due to selective publication. This showcases the situation where more studies are likely to be unpublished compared to the $p = 0.7$.

We summarized the sparsity of simulated datasets in Web Table 11 and the resulting SAUC in Web Table 12. The complete results are summarized in Web Table 13. The average of SAUCs under our proposed method was closer to the true values compared with the method by Zhou et al.[19], suggesting the advantage of the bivariate binomial model in sparse meta-analysis. In addition, the estimate by our proposed method in Table 4 in the main body and Web Table 11 are very close. It validated the robustness of our proposed method across various marginal selection probabilities.

WEB TABLE 8 Summary of the estimates under the true selection mechanism of $(c_0, c_1) = (0, 1)$ given $p \approx 0.7$. (The estimates are summarized by mean (empirical standard error) over 1000 simulated meta-analyses; – for θ, α within the results for Zhou et al.[19] is because the θ, α is not available for the bivariate normal model)

Experiment		True	$S = 15$			$S = 25$			$S = 50$			$S = 100$		
			Our proposal	Bivariate binomial with published studies	Method of Zhou et al.[19]	Our proposal	Bivariate binomial with published studies	Method of Zhou et al.[19]	Our proposal	Bivariate binomial with published studies	Method of Zhou et al.[19]	Our proposal	Bivariate binomial with published studies	Method of Zhou et al.[19]
1	Sensitivity	0.900	0.896 (0.038)	0.897 (0.034)	0.860 (0.039)	0.896 (0.029)	0.898 (0.025)	0.860 (0.031)	0.897 (0.022)	0.898 (0.018)	0.860 (0.023)	0.900 (0.017)	0.900 (0.014)	0.864 (0.017)
	Specificity	0.500	0.529 (0.074)	0.602 (0.061)	0.516 (0.072)	0.523 (0.064)	0.606 (0.049)	0.510 (0.058)	0.515 (0.049)	0.610 (0.034)	0.506 (0.041)	0.510 (0.036)	0.611 (0.023)	0.501 (0.029)
	θ	1.184	1.196 (0.529)	0.943 (0.514)	-	1.182 (0.461)	0.895 (0.462)	-	1.126 (0.285)	0.786 (0.257)	-	1.142 (0.174)	0.772 (0.133)	-
	α	2.368	2.625 (0.976)	2.764 (0.823)	-	2.549 (0.871)	2.712 (0.759)	-	2.386 (0.492)	2.558 (0.391)	-	2.370 (0.275)	2.530 (0.209)	-
	SAUC	0.832	0.823 (0.065)	0.839 (0.057)	0.816 (0.059)	0.825 (0.054)	0.842 (0.047)	0.819 (0.049)	0.824 (0.038)	0.841 (0.032)	0.820 (0.034)	0.829 (0.026)	0.844 (0.021)	0.825 (0.024)
2	Sensitivity	0.500	0.499 (0.072)	0.498 (0.069)	0.499 (0.063)	0.500 (0.057)	0.500 (0.056)	0.500 (0.050)	0.499 (0.041)	0.500 (0.039)	0.500 (0.035)	0.508 (0.028)	0.508 (0.027)	0.507 (0.024)
	Specificity	0.900	0.907 (0.023)	0.914 (0.016)	0.909 (0.018)	0.905 (0.017)	0.914 (0.012)	0.910 (0.012)	0.904 (0.011)	0.915 (0.008)	0.910 (0.009)	0.903 (0.007)	0.915 (0.005)	0.910 (0.005)
	θ	-1.019	-1.187 (0.394)	-1.403 (0.416)	-	-1.124 (0.257)	-1.389 (0.286)	-	-1.109 (0.163)	-1.403 (0.189)	-	-1.076 (0.093)	-1.392 (0.114)	-
	α	2.038	2.358 (0.829)	2.787 (0.872)	-	2.254 (0.510)	2.778 (0.567)	-	2.210 (0.341)	2.803 (0.385)	-	2.219 (0.211)	2.840 (0.250)	-
	SAUC	0.798	0.808 (0.079)	0.843 (0.068)	0.813 (0.086)	0.811 (0.055)	0.854 (0.044)	0.825 (0.059)	0.812 (0.037)	0.862 (0.026)	0.834 (0.036)	0.816 (0.022)	0.867 (0.015)	0.840 (0.021)
3	Sensitivity	0.800	0.793 (0.053)	0.793 (0.049)	0.762 (0.048)	0.798 (0.042)	0.799 (0.039)	0.766 (0.038)	0.797 (0.030)	0.798 (0.028)	0.766 (0.027)	0.798 (0.022)	0.799 (0.020)	0.766 (0.018)
	Specificity	0.800	0.825 (0.043)	0.854 (0.033)	0.833 (0.046)	0.826 (0.034)	0.858 (0.025)	0.837 (0.036)	0.826 (0.024)	0.859 (0.017)	0.841 (0.025)	0.829 (0.019)	0.863 (0.012)	0.845 (0.020)
	θ	0.104	-0.007 (0.491)	-0.290 (0.506)	-	0.009 (0.395)	-0.321 (0.417)	-	-0.025 (0.231)	-0.395 (0.235)	-	-0.014 (0.170)	-0.403 (0.170)	-
	α	2.780	3.058 (0.529)	3.308 (0.503)	-	3.041 (0.416)	3.331 (0.394)	-	2.970 (0.268)	3.293 (0.259)	-	2.967 (0.190)	3.303 (0.186)	-
	SAUC	0.869	0.871 (0.037)	0.888 (0.032)	0.854 (0.044)	0.877 (0.029)	0.896 (0.023)	0.862 (0.035)	0.880 (0.021)	0.900 (0.015)	0.870 (0.024)	0.882 (0.015)	0.903 (0.010)	0.873 (0.016)
4	Sensitivity	0.900	0.870 (0.054)	0.842 (0.052)	0.856 (0.047)	0.872 (0.042)	0.843 (0.039)	0.860 (0.036)	0.879 (0.032)	0.843 (0.029)	0.866 (0.027)	0.875 (0.022)	0.841 (0.021)	0.868 (0.018)
	Specificity	0.500	0.574 (0.108)	0.649 (0.086)	0.532 (0.103)	0.571 (0.089)	0.653 (0.065)	0.520 (0.085)	0.560 (0.072)	0.656 (0.047)	0.507 (0.060)	0.568 (0.055)	0.659 (0.034)	0.495 (0.045)
	θ	1.184	0.937 (0.482)	0.612 (0.445)	-	0.924 (0.364)	0.589 (0.318)	-	0.950 (0.289)	0.559 (0.222)	-	0.925 (0.206)	0.556 (0.159)	-
	α	2.368	2.458 (0.493)	2.461 (0.435)	-	2.404 (0.302)	2.419 (0.285)	-	2.369 (0.185)	2.390 (0.176)	-	2.365 (0.124)	2.388 (0.117)	-
	SAUC	0.832	0.828 (0.033)	0.830 (0.032)	0.815 (0.032)	0.830 (0.024)	0.832 (0.023)	0.815 (0.023)	0.830 (0.017)	0.833 (0.016)	0.816 (0.016)	0.831 (0.011)	0.834 (0.011)	0.816 (0.011)
5	Sensitivity	0.500	0.460 (0.087)	0.443 (0.076)	0.458 (0.071)	0.469 (0.072)	0.446 (0.060)	0.462 (0.056)	0.472 (0.047)	0.444 (0.041)	0.459 (0.037)	0.474 (0.032)	0.443 (0.030)	0.460 (0.027)
	Specificity	0.900	0.913 (0.027)	0.920 (0.019)	0.913 (0.022)	0.912 (0.023)	0.921 (0.016)	0.914 (0.018)	0.911 (0.015)	0.922 (0.010)	0.915 (0.011)	0.911 (0.010)	0.923 (0.006)	0.916 (0.007)
	θ	-1.019	-1.243 (0.376)	-1.385 (0.335)	-	-1.209 (0.303)	-1.383 (0.262)	-	-1.186 (0.193)	-1.397 (0.176)	-	-1.178 (0.145)	-1.405 (0.130)	-
	α	2.038	2.114 (0.564)	2.292 (0.584)	-	2.134 (0.399)	2.330 (0.421)	-	2.130 (0.273)	2.352 (0.293)	-	2.141 (0.174)	2.367 (0.187)	-
	SAUC	0.798	0.795 (0.066)	0.813 (0.061)	0.792 (0.067)	0.802 (0.046)	0.822 (0.041)	0.799 (0.048)	0.805 (0.031)	0.828 (0.028)	0.804 (0.033)	0.808 (0.019)	0.831 (0.017)	0.807 (0.020)
6	Sensitivity	0.800	0.748 (0.077)	0.717 (0.070)	0.724 (0.074)	0.748 (0.058)	0.711 (0.053)	0.716 (0.057)	0.755 (0.045)	0.711 (0.041)	0.716 (0.044)	0.761 (0.028)	0.712 (0.026)	0.717 (0.029)
	Specificity	0.800	0.843 (0.058)	0.873 (0.039)	0.842 (0.063)	0.846 (0.043)	0.878 (0.027)	0.850 (0.045)	0.843 (0.034)	0.879 (0.020)	0.853 (0.034)	0.843 (0.022)	0.881 (0.013)	0.856 (0.022)
	θ	0.104	-0.209 (0.477)	-0.478 (0.431)	-	-0.210 (0.346)	-0.518 (0.314)	-	-0.181 (0.251)	-0.534 (0.217)	-	-0.183 (0.157)	-0.564 (0.127)	-
	α	2.780	2.877 (0.367)	2.947 (0.371)	-	2.833 (0.257)	2.918 (0.266)	-	2.815 (0.184)	2.910 (0.191)	-	2.826 (0.132)	2.927 (0.132)	-
	SAUC	0.869	0.867 (0.034)	0.873 (0.032)	0.853 (0.036)	0.868 (0.022)	0.876 (0.021)	0.854 (0.026)	0.870 (0.016)	0.878 (0.015)	0.856 (0.018)	0.872 (0.012)	0.881 (0.010)	0.858 (0.012)

WEB TABLE 9 Summary of the sparsity of simulated datasets under $(c_0, c_1) = (1, 0)$ given $p \approx 0.7$. Full indicates published and unpublished studies; Published indicates published studies.

Experiment	Rate(%)	S=15		S=25		S=50		S=100	
		Full	Published	Full	Published	Full	Published	Full	Published
1	Zero entries	18.8	19.4	18.9	19.5	19.3	19.7	20.0	20.2
	No-more-than-3-entries	80.8	75.1	81.0	75.1	81.7	75.6	81.4	75.8
	No-more-than-5-entries	94.5	90.0	94.9	89.9	95.1	90.1	95.4	90.6
2	Zero entries	0.5	0.7	0.7	0.8	0.7	0.9	0.8	1.0
	No-more-than-3-entries	15.7	18.3	15.2	18.4	14.5	18.2	15.1	18.3
	No-more-than-5-entries	39.3	42.9	39.1	43.1	37.8	42.4	38.2	42.7
3	Zero entries	8.4	6.7	8.7	6.9	8.5	6.7	8.5	6.5
	No-more-than-3-entries	59.4	47.6	59.0	46.9	59.5	47.0	58.8	46.0
	No-more-than-5-entries	81.7	70.3	82.4	70.2	82.5	70.3	82.7	70.2
4	Zero entries	25.2	23.2	25.6	23.4	25.1	23.0	24.2	22.3
	No-more-than-3-entries	80.5	70.6	81.3	71.1	81.5	70.9	81.0	70.7
	No-more-than-5-entries	93.3	85.0	93.9	85.5	93.8	85.0	93.9	85.4
5	Zero entries	2.6	3.6	2.5	3.8	2.3	3.7	2.6	4.1
	No-more-than-3-entries	24.3	30.5	23.7	30.6	23.4	30.3	23.5	30.9
	No-more-than-5-entries	47.8	54.0	47.0	54.0	46.6	53.9	46.3	54.0
6	Zero entries	13.6	10.6	14.2	11.1	14.2	10.8	14.4	10.9
	No-more-than-3-entries	62.8	50.8	64.2	51.5	63.8	50.7	65.0	51.6
	No-more-than-5-entries	82.6	71.8	83.4	72.1	83.3	71.6	83.8	71.7

Web Appendix I | SIMULATION STUDIES UNDER MISSPECIFIED MARGINAL SELECTION PROBABILITY

The marginal selection probability plays an important role in providing unbiased estimations of SAUC and other key parameters of interest. However, it is unknown in practice. To see what would happen if the marginal selection probability is misspecified, we generated the simulated meta-analyses under the settings in Section 5 while estimating the SAUC with our proposed method and the sensitivity analysis method based on the bivariate normal model proposed by Zhou et al.[19] under $p = 0.5$, which is different from the true value. We summarized the SAUC estimations in Web Table 14. We can see that the estimates contain unignorable bias under misspecified p, however, an interesting finding is that our proposed method can still obtain less biased estimations compared to the method by Zhou et al.[19] in most cases except Scenario 1, indicating that the proposed method is more suitable for sparse diagnostic meta-analyses even when the marginal selection probability is misspecified.

WEB TABLE 10 Summary of the estimates under the true selection mechanism of $(c_0, c_1) = (1, 0)$ given $p \approx 0.7$. (The estimates are summarized by mean (empirical standard error) over 1000 simulated meta-analyses; – for θ, α within the results for Zhou et al.[19] is because the θ, α is not available for the bivariate normal model)

Experiment		True	S = 15			S = 25			S = 50			S = 100		
			Our proposal	Bivariate binomial with published studies	Method of Zhou et al.[19]	Our proposal	Bivariate binomial with published studies	Method of Zhou et al.[19]	Our proposal	Bivariate binomial with published studies	Method of Zhou et al.[19]	Our proposal	Bivariate binomial with published studies	Method of Zhou et al.[19]
1	Sensitivity	0.900	0.917 (0.024)	0.909 (0.026)	0.875 (0.028)	0.918 (0.018)	0.909 (0.020)	0.873 (0.022)	0.920 (0.011)	0.911 (0.014)	0.874 (0.015)	0.922 (0.008)	0.911 (0.011)	0.874 (0.011)
	Specificity	0.500	0.497 (0.070)	0.497 (0.070)	0.498 (0.070)	0.499 (0.057)	0.499 (0.055)	0.499 (0.054)	0.499 (0.039)	0.499 (0.039)	0.499 (0.039)	0.502 (0.028)	0.502 (0.027)	0.502 (0.027)
	θ	1.184	1.647 (0.534)	1.973 (0.565)	-	1.585 (0.493)	2.000 (0.583)	-	1.512 (0.298)	1.942 (0.538)	-	1.483 (0.205)	1.828 (0.456)	-
	α	2.368	3.277 (1.064)	3.937 (1.107)	-	3.165 (0.965)	4.002 (1.150)	-	3.019 (0.603)	3.880 (1.080)	-	2.975 (0.436)	3.662 (0.925)	-
	SAUC	0.832	0.869 (0.048)	0.882 (0.037)	0.857 (0.039)	0.870 (0.038)	0.884 (0.030)	0.859 (0.028)	0.872 (0.028)	0.886 (0.023)	0.864 (0.021)	0.874 (0.019)	0.886 (0.015)	0.866 (0.013)
2	Sensitivity	0.500	0.550 (0.072)	0.598 (0.063)	0.526 (0.075)	0.551 (0.055)	0.601 (0.051)	0.530 (0.066)	0.541 (0.041)	0.600 (0.036)	0.528 (0.050)	0.546 (0.032)	0.606 (0.023)	0.546 (0.035)
	Specificity	0.900	0.896 (0.031)	0.897 (0.028)	0.893 (0.032)	0.897 (0.023)	0.898 (0.021)	0.894 (0.023)	0.898 (0.016)	0.899 (0.014)	0.895 (0.016)	0.898 (0.010)	0.899 (0.010)	0.895 (0.010)
	θ	-1.019	-0.737 (0.372)	-0.441 (0.409)	-	-0.725 (0.315)	-0.435 (0.345)	-	-0.773 (0.226)	-0.464 (0.233)	-	-0.768 (0.177)	-0.468 (0.144)	-
	α	2.038	2.021 (0.673)	2.161 (0.587)	-	2.023 (0.478)	2.162 (0.433)	-	1.982 (0.321)	2.133 (0.302)	-	2.015 (0.195)	2.168 (0.196)	-
	SAUC	0.798	0.766 (0.084)	0.768 (0.080)	0.736 (0.091)	0.774 (0.065)	0.774 (0.066)	0.740 (0.075)	0.777 (0.045)	0.779 (0.047)	0.743 (0.058)	0.785 (0.027)	0.788 (0.030)	0.747 (0.034)
3	Sensitivity	0.800	0.846 (0.051)	0.846 (0.038)	0.800 (0.046)	0.854 (0.039)	0.847 (0.028)	0.800 (0.034)	0.864 (0.027)	0.848 (0.020)	0.799 (0.024)	0.871 (0.016)	0.847 (0.014)	0.799 (0.016)
	Specificity	0.800	0.797 (0.046)	0.797 (0.045)	0.794 (0.046)	0.800 (0.037)	0.800 (0.036)	0.798 (0.036)	0.799 (0.025)	0.799 (0.025)	0.796 (0.025)	0.798 (0.018)	0.799 (0.017)	0.796 (0.018)
	θ	0.104	0.594 (0.557)	1.039 (0.601)	-	0.482 (0.487)	1.045 (0.568)	-	0.357 (0.386)	0.962 (0.479)	-	0.281 (0.269)	0.927 (0.369)	-
	α	2.780	3.473 (0.620)	3.857 (0.736)	-	3.432 (0.478)	3.854 (0.694)	-	3.365 (0.358)	3.719 (0.573)	-	3.332 (0.262)	3.646 (0.421)	-
	SAUC	0.869	0.885 (0.042)	0.882 (0.034)	0.840 (0.041)	0.893 (0.032)	0.884 (0.026)	0.839 (0.032)	0.900 (0.022)	0.886 (0.019)	0.835 (0.024)	0.905 (0.013)	0.887 (0.014)	0.832 (0.022)
4	Sensitivity	0.900	0.926 (0.026)	0.922 (0.029)	0.884 (0.031)	0.928 (0.019)	0.923 (0.022)	0.886 (0.024)	0.926 (0.013)	0.922 (0.015)	0.885 (0.017)	0.925 (0.009)	0.921 (0.010)	0.884 (0.012)
	Specificity	0.500	0.427 (0.088)	0.430 (0.088)	0.451 (0.090)	0.426 (0.068)	0.429 (0.069)	0.450 (0.071)	0.425 (0.047)	0.429 (0.048)	0.449 (0.049)	0.426 (0.032)	0.430 (0.033)	0.450 (0.036)
	θ	1.184	1.686 (0.441)	1.762 (0.464)	-	1.698 (0.347)	1.769 (0.386)	-	1.669 (0.208)	1.718 (0.219)	-	1.663 (0.148)	1.704 (0.154)	-
	α	2.368	2.839 (0.742)	3.029 (0.862)	-	2.885 (0.580)	3.073 (0.718)	-	2.833 (0.325)	2.978 (0.393)	-	2.835 (0.234)	2.969 (0.272)	-
	SAUC	0.832	0.855 (0.043)	0.860 (0.041)	0.846 (0.038)	0.863 (0.031)	0.868 (0.029)	0.850 (0.029)	0.864 (0.020)	0.869 (0.019)	0.849 (0.019)	0.865 (0.013)	0.870 (0.013)	0.850 (0.013)
5	Sensitivity	0.500	0.593 (0.108)	0.628 (0.086)	0.526 (0.104)	0.591 (0.090)	0.632 (0.068)	0.525 (0.086)	0.590 (0.071)	0.637 (0.048)	0.529 (0.070)	0.580 (0.052)	0.641 (0.030)	0.533 (0.050)
	Specificity	0.900	0.855 (0.063)	0.840 (0.058)	0.875 (0.054)	0.861 (0.047)	0.843 (0.043)	0.881 (0.039)	0.864 (0.036)	0.843 (0.030)	0.882 (0.030)	0.871 (0.024)	0.842 (0.019)	0.883 (0.021)
	θ	-1.019	-0.593 (0.440)	-0.349 (0.439)	-	-0.613 (0.348)	-0.355 (0.339)	-	-0.610 (0.263)	-0.338 (0.243)	-	-0.648 (0.169)	-0.326 (0.152)	-
	α	2.038	2.056 (0.388)	2.086 (0.345)	-	2.070 (0.309)	2.091 (0.258)	-	2.059 (0.227)	2.087 (0.174)	-	2.037 (0.176)	2.084 (0.127)	-
	SAUC	0.798	0.789 (0.049)	0.787 (0.045)	0.768 (0.048)	0.794 (0.038)	0.792 (0.034)	0.772 (0.037)	0.794 (0.028)	0.793 (0.023)	0.772 (0.025)	0.793 (0.021)	0.795 (0.017)	0.773 (0.018)
6	Sensitivity	0.800	0.870 (0.049)	0.863 (0.044)	0.810 (0.059)	0.879 (0.033)	0.868 (0.032)	0.816 (0.041)	0.883 (0.020)	0.868 (0.023)	0.815 (0.031)	0.888 (0.013)	0.870 (0.017)	0.818 (0.023)
	Specificity	0.800	0.711 (0.080)	0.713 (0.076)	0.740 (0.080)	0.706 (0.060)	0.711 (0.057)	0.737 (0.060)	0.705 (0.042)	0.713 (0.042)	0.741 (0.045)	0.701 (0.030)	0.711 (0.032)	0.737 (0.033)
	θ	0.104	0.688 (0.474)	0.863 (0.486)	-	0.745 (0.339)	0.910 (0.355)	-	0.734 (0.238)	0.870 (0.243)	-	0.761 (0.171)	0.890 (0.178)	-
	α	2.780	3.079 (0.435)	3.220 (0.551)	-	3.090 (0.317)	3.232 (0.405)	-	3.067 (0.196)	3.172 (0.250)	-	3.077 (0.138)	3.178 (0.189)	-
	SAUC	0.869	0.882 (0.026)	0.880 (0.025)	0.853 (0.029)	0.886 (0.019)	0.883 (0.019)	0.854 (0.022)	0.888 (0.013)	0.884 (0.013)	0.853 (0.016)	0.890 (0.009)	0.885 (0.009)	0.854 (0.012)

WEB TABLE 11 Summary of the sparsity of simulated datasets under $(c_0, c_1) = (1/\sqrt{2}, 1/\sqrt{2})$ given $p \approx 0.5$. Full indicates published and unpublished studies; Published indicates published studies.

Experiment	Rate (%)	$S = 15$		$S = 25$		$S = 50$	
		Full	Published	Full	Published	Full	Published
1	Zero entries	17.9	19.6	18.6	20.6	19.4	20.0
	No-more-than-3-entries	81.4	76.0	79.3	76.0	79.9	75.0
	No-more-than-5-entries	93.7	89.8	93.7	90.8	93.6	90.1
2	Zero entries	0.2	0.3	0.6	0.8	0.3	0.7
	No-more-than-3-entries	18.3	18.5	14.7	18.2	15.5	18.6
	No-more-than-5-entries	37.1	41.5	37.2	42.6	36.2	41.2
3	Zero entries	3.2	6.5	2.6	7.2	3.3	6.8
	No-more-than-3-entries	42.8	46.9	47.3	47.7	46.6	46.3
	No-more-than-5-entries	71.1	70.1	71.2	69.8	71.2	69.3
4	Zero entries	10.7	23.8	10.5	24.9	9.7	23.4
	No-more-than-3-entries	62.5	75.2	58.1	72.4	55.1	70.8
	No-more-than-5-entries	79.3	87.3	77.7	86.2	75.2	84.7
5	Zero entries	0.8	3.3	0.6	3.0	0.5	3.7
	No-more-than-3-entries	17.0	30.7	16.9	29.5	17.7	29.9
	No-more-than-5-entries	40.3	53.9	39.5	53.2	39.0	53.0
6	Zero entries	1.0	9.8	1.2	10.5	1.2	11.1
	No-more-than-3-entries	32.9	50.4	32.4	51.0	33.2	51.9
	No-more-than-5-entries	57.3	70.7	56.1	70.1	58.2	71.6

WEB TABLE 12 Summary of the SAUC estimates under the true selection mechanism of $(c_0, c_1) = (1/\sqrt{2}, 1/\sqrt{2})$ given $p \approx 0.5$. (The estimates are summarized by mean (empirical standard error) over 1000 simulated meta-analyses; the values are multiplied by 100.)

Experiment	Method	TRUE	$S = 15$	$S = 25$	$S = 50$
			AVE(SD)	AVE(SD)	AVE(SD)
	MLE with published studies		88.8 (4.3)	88.4 (3.6)	89.1 (2.2)
	Method of Zhou et al.[19 19]		83.8 (4.7)	84.1 (3.4)	84.6 (2.6)
1	Proposal with $(c_0, c_1) = (1/\sqrt{2}, 1/\sqrt{2})$	83.2	84.1 (6.7)	83.1 (5.7)	83.9 (4.2)
	Proposal with $(c_0, c_1) = (1, 0)$		87.1 (5.5)	86.3 (4.3)	87.0 (3.0)
	Proposal with $(c_0, c_1) = (0, 1)$		87.0 (6.0)	87.4 (3.6)	88.1 (2.9)
	MLE with published studies		84.4 (7.4)	84.2 (6.1)	84.8 (5.2)
	Method of Zhou et al.[19 19]		78.3 (9.9)	77.2 (8.5)	77.4 (7.9)
2	Proposal with $(c_0, c_1) = (1/\sqrt{2}, 1/\sqrt{2})$	79.8	81.0 (9.2)	79.6 (8.3)	79.3 (8.0)
	Proposal with $(c_0, c_1) = (1, 0)$		84.5 (7.6)	84.5 (6.2)	84.8 (4.8)
	Proposal with $(c_0, c_1) = (0, 1)$		80.6 (8.1)	79.5 (6.2)	79.1 (5.4)
	MLE with published studies		88.8 (3.8)	89.7 (2.8)	90.1 (1.9)
	Method of Zhou et al.[19 19]		83.8 (5.1)	84.1 (4.6)	84.5 (3.7)
3	Proposal with $(c_0, c_1) = (1/\sqrt{2}, 1/\sqrt{2})$	86.9	86.9 (5.6)	88.1 (4.6)	87.9 (3.6)
	Proposal with $(c_0, c_1) = (1, 0)$		89.2 (4.2)	90.4 (3.2)	90.7 (2.1)
	Proposal with $(c_0, c_1) = (0, 1)$		86.5 (4.1)	87.2 (3.3)	87.4 (2.2)
	MLE with published studies		85.0 (3.2)	85.0 (2.4)	84.8 (1.6)
	Method of Zhou et al.[19 19]		80.9 (4.0)	80.1 (2.9)	79.9 (2.2)
4	Proposal with $(c_0, c_1) = (1/\sqrt{2}, 1/\sqrt{2})$	83.2	83.2 (4.7)	83.3 (3.7)	83.3 (2.9)
	Proposal with $(c_0, c_1) = (1, 0)$		84.6 (3.7)	84.7 (2.7)	84.7 (1.9)
	Proposal with $(c_0, c_1) = (0, 1)$		84.9 (3.2)	84.8 (2.4)	84.6 (1.6)
	MLE with published studies		81.1 (6.5)	82.8 (3.5)	82.3 (2.8)
	Method of Zhou et al.[19 19]		75.9 (8.7)	77.6 (5.0)	77.0 (4.0)
5	Proposal with $(c_0, c_1) = (1/\sqrt{2}, 1/\sqrt{2})$	79.8	79.0 (9.1)	81.1 (5.4)	80.7 (5.5)
	Proposal with $(c_0, c_1) = (1, 0)$		80.9 (6.7)	82.4 (3.7)	82.0 (2.9)
	Proposal with $(c_0, c_1) = (0, 1)$		79.6 (7.5)	81.7 (3.5)	81.3 (2.9)
	MLE with published studies		87.4 (3.4)	87.5 (2.8)	87.9 (1.6)
	Method of Zhou et al.[19 19]		82.5 (4.9)	82.9 (4.1)	83.3 (2.7)
6	Proposal with $(c_0, c_1) = (1/\sqrt{2}, 1/\sqrt{2})$	86.9	86.6 (5.2)	87.0 (4.6)	87.6 (3.4)
	Proposal with $(c_0, c_1) = (1, 0)$		87.7 (3.5)	87.7 (2.8)	88.0 (1.6)
	Proposal with $(c_0, c_1) = (0, 1)$		86.5 (3.9)	86.7 (2.9)	87.0 (1.8)

WEB TABLE 13 Summary of the estimates under the true selection mechanism of $(c_0, c_1) = (1/\sqrt{2}, 1/\sqrt{2})$ given $p \approx 0.5$. (The estimates are summarized by mean (empirical standard error) over 1000 simulated meta-analyses; – for θ, α within the results for Zhou et al.[19] is because the θ, α is not available for the bivariate normal model)

Experiment		True	$S = 15$ Our proposal	$S = 15$ MLE with published studies	$S = 15$ Method of Zhou et al.[19]	$S = 25$ Our proposal	$S = 25$ MLE with published studies	$S = 25$ Method of Zhou et al.[19]	$S = 50$ Our proposal	$S = 50$ MLE with published studies	$S = 50$ Method of Zhou et al.[19]
1	Sensitivity	0.900	0.927 (0.044)	0.909 (0.032)	0.855 (0.038)	0.933 (0.040)	0.908 (0.023)	0.849 (0.026)	0.939 (0.038)	0.908 (0.018)	0.849 (0.021)
	Specificity	0.500	0.564 (0.096)	0.619 (0.063)	0.565 (0.077)	0.585 (0.075)	0.624 (0.053)	0.577 (0.064)	0.607 (0.047)	0.641 (0.036)	0.591 (0.045)
	θ	1.184	1.090 (0.591)	1.496 (0.730)	-	0.876 (0.508)	1.297 (0.579)	-	0.773 (0.387)	1.236 (0.502)	-
	α	2.368	2.922 (0.850)	3.784 (1.166)	-	2.786 (0.740)	3.453 (0.937)	-	2.798 (0.657)	3.445 (0.776)	-
	SAUC	0.832	0.841 (0.067)	0.888 (0.043)	0.838 (0.047)	0.831 (0.057)	0.884 (0.036)	0.841 (0.034)	0.839 (0.042)	0.891 (0.022)	0.846 (0.026)
2	Sensitivity	0.500	0.537 (0.093)	0.618 (0.072)	0.556 (0.080)	0.532 (0.081)	0.614 (0.054)	0.555 (0.058)	0.511 (0.056)	0.604 (0.044)	0.549 (0.044)
	Specificity	0.900	0.906 (0.036)	0.930 (0.019)	0.907 (0.031)	0.900 (0.030)	0.929 (0.017)	0.905 (0.024)	0.903 (0.028)	0.935 (0.011)	0.910 (0.021)
	θ	-1.019	-1.029 (0.695)	-0.805 (0.516)	-	-0.929 (0.562)	-0.758 (0.354)	-	-1.025 (0.524)	-0.843 (0.258)	-
	α	2.038	2.550 (0.980)	2.867 (0.743)	-	2.298 (0.746)	2.721 (0.510)	-	2.240 (0.752)	2.728 (0.467)	-
	SAUC	0.798	0.810 (0.092)	0.844 (0.074)	0.783 (0.099)	0.796 (0.083)	0.842 (0.061)	0.772 (0.085)	0.793 (0.080)	0.848 (0.052)	0.774 (0.079)
3	Sensitivity	0.800	0.802 (0.079)	0.806 (0.048)	0.754 (0.051)	0.806 (0.077)	0.807 (0.036)	0.753 (0.040)	0.809 (0.070)	0.810 (0.023)	0.756 (0.023)
	Specificity	0.800	0.826 (0.060)	0.870 (0.034)	0.841 (0.045)	0.828 (0.048)	0.873 (0.026)	0.847 (0.034)	0.827 (0.032)	0.870 (0.017)	0.846 (0.021)
	θ	0.104	0.018 (0.749)	0.250 (0.617)	-	-0.271 (0.552)	0.149 (0.524)	-	-0.365 (0.468)	0.033 (0.335)	-
	α	2.780	3.340 (0.879)	3.547 (0.602)	-	3.291 (0.802)	3.497 (0.482)	-	3.273 (0.694)	3.394 (0.227)	-
	SAUC	0.869	0.869 (0.056)	0.888 (0.038)	0.838 (0.051)	0.881 (0.046)	0.897 (0.028)	0.841 (0.046)	0.879 (0.036)	0.901 (0.019)	0.845 (0.037)
4	Sensitivity	0.900	0.901 (0.065)	0.854 (0.054)	0.806 (0.051)	0.904 (0.068)	0.843 (0.049)	0.791 (0.049)	0.909 (0.056)	0.834 (0.036)	0.782 (0.031)
	Specificity	0.500	0.572 (0.116)	0.673 (0.096)	0.622 (0.111)	0.588 (0.092)	0.686 (0.069)	0.629 (0.086)	0.603 (0.061)	0.705 (0.047)	0.651 (0.057)
	θ	1.184	0.937 (0.634)	0.656 (0.514)	-	0.823 (0.513)	0.559 (0.389)	-	0.741 (0.349)	0.456 (0.256)	-
	α	2.368	2.650 (0.636)	2.729 (0.519)	-	2.559 (0.424)	2.624 (0.358)	-	2.549 (0.341)	2.574 (0.220)	-
	SAUC	0.832	0.832 (0.047)	0.850 (0.032)	0.809 (0.040)	0.833 (0.037)	0.850 (0.024)	0.801 (0.029)	0.833 (0.029)	0.848 (0.016)	0.799 (0.022)
5	Sensitivity	0.500	0.498 (0.140)	0.552 (0.088)	0.522 (0.087)	0.484 (0.130)	0.560 (0.069)	0.530 (0.069)	0.462 (0.099)	0.559 (0.047)	0.535 (0.048)
	Specificity	0.900	0.899 (0.054)	0.912 (0.026)	0.884 (0.038)	0.898 (0.060)	0.912 (0.025)	0.881 (0.040)	0.905 (0.048)	0.912 (0.015)	0.884 (0.022)
	θ	-1.019	-1.099 (0.639)	-0.917 (0.343)	-	-1.168 (0.681)	-0.920 (0.300)	-	-1.225 (0.569)	-0.893 (0.193)	-
	α	2.038	2.166 (0.796)	2.293 (0.537)	-	2.289 (0.534)	2.390 (0.327)	-	2.211 (0.440)	2.333 (0.233)	-
	SAUC	0.798	0.790 (0.091)	0.811 (0.065)	0.759 (0.087)	0.811 (0.054)	0.828 (0.035)	0.776 (0.050)	0.807 (0.055)	0.823 (0.028)	0.770 (0.040)
6	Sensitivity	0.800	0.748 (0.125)	0.725 (0.067)	0.681 (0.071)	0.777 (0.098)	0.726 (0.049)	0.685 (0.048)	0.780 (0.091)	0.724 (0.037)	0.681 (0.039)
	Specificity	0.800	0.834 (0.080)	0.883 (0.041)	0.853 (0.056)	0.822 (0.066)	0.884 (0.032)	0.858 (0.042)	0.827 (0.041)	0.887 (0.024)	0.863 (0.026)
	θ	0.104	-0.306 (0.731)	-0.370 (0.449)	-	-0.151 (0.618)	-0.354 (0.325)	-	-0.209 (0.476)	-0.393 (0.244)	-
	α	2.780	3.046 (0.603)	3.012 (0.373)	-	3.027 (0.532)	2.967 (0.287)	-	3.010 (0.387)	2.963 (0.158)	-
	SAUC	0.869	0.866 (0.052)	0.874 (0.034)	0.825 (0.049)	0.870 (0.046)	0.875 (0.028)	0.829 (0.041)	0.876 (0.034)	0.879 (0.016)	0.833 (0.027)

WEB TABLE 14 Summary of the SAUC estimates under the true selection mechanism of $(c_0, c_1) = (1/\sqrt{2}, 1/\sqrt{2})$ under misspecified p. (The estimates are summarized by mean (empirical standard error) over 1000 simulated meta-analyses; the values are multiplied by 100.)

Experiment	Method	TRUE	S=15 AVE(SD)	S=25 AVE(SD)	S=50 AVE(SD)
1	MLE with published studies	83.2	88.1 (4.3)	87.4 (3.5)	87.7 (2.3)
	Method of Zhou et al. [19]		82.5 (4.9)	82.9 (3.6)	82.7 (3.3)
	Proposal with $(c_0, c_1) = (1/\sqrt{2}, 1/\sqrt{2})$		81.4 (7.2)	81.2 (5.3)	80.6 (4.4)
	Proposal with $(c_0, c_1) = (1, 0)$		85.7 (4.8)	85.0 (4.0)	85.2 (2.8)
	Proposal with $(c_0, c_1) = (0, 1)$		86.8 (5.0)	86.6 (3.7)	87.0 (2.7)
2	MLE with published studies	79.8	81.2 (7.4)	83.0 (5.2)	83.4 (3.6)
	Method of Zhou et al. [19]		72.0 (9.5)	74.2 (8.1)	73.8 (5.6)
	Proposal with $(c_0, c_1) = (1/\sqrt{2}, 1/\sqrt{2})$		74.3 (10.1)	75.8 (8.6)	74.6 (6.2)
	Proposal with $(c_0, c_1) = (1, 0)$		81.1 (7.2)	82.9 (5.4)	82.9 (3.6)
	Proposal with $(c_0, c_1) = (0, 1)$		75.6 (7.3)	76.8 (6.3)	75.9 (4.3)
3	MLE with published studies	86.9	88.3 (3.6)	89.2 (2.6)	89.7 (1.5)
	Method of Zhou et al. [19]		81.7 (4.7)	82.0 (4.4)	83.1 (2.9)
	Proposal with $(c_0, c_1) = (1/\sqrt{2}, 1/\sqrt{2})$		85.1 (5.7)	85.8 (5.6)	86.2 (4.5)
	Proposal with $(c_0, c_1) = (1, 0)$		88.7 (4.2)	89.9 (3.3)	90.3 (2.5)
	Proposal with $(c_0, c_1) = (0, 1)$		85.2 (5.9)	86.4 (2.8)	86.8 (2.2)
4	MLE with published studies	83.2	84.8 (3.1)	84.3 (2.3)	84.5 (1.4)
	Method of Zhou et al. [19]		80.7 (3.7)	79.4 (2.6)	79.4 (1.7)
	Proposal with $(c_0, c_1) = (1/\sqrt{2}, 1/\sqrt{2})$		82.5 (4.1)	81.8 (3.6)	82.6 (2.4)
	Proposal with $(c_0, c_1) = (1, 0)$		84.4 (3.6)	84.1 (2.2)	84.3 (1.6)
	Proposal with $(c_0, c_1) = (0, 1)$		84.7 (3.1)	84.2 (2.3)	84.3 (1.4)
5	MLE with published studies	79.8	81.1 (4.1)	81.1 (3.3)	81.2 (2.1)
	Method of Zhou et al. [19]		75.5 (5.6)	74.8 (4.7)	74.7 (3.0)
	Proposal with $(c_0, c_1) = (1/\sqrt{2}, 1/\sqrt{2})$		78.4 (6.8)	78.5 (6.0)	78.5 (4.9)
	Proposal with $(c_0, c_1) = (1, 0)$		80.8 (4.1)	80.8 (3.5)	80.9 (2.2)
	Proposal with $(c_0, c_1) = (0, 1)$		80.0 (4.3)	80.0 (3.4)	80.2 (2.2)
6	MLE with published studies	86.9	87.2 (2.3)	87.5 (2.2)	87.6 (1.2)
	Method of Zhou et al. [19]		81.9 (4.3)	82.3 (3.0)	82.3 (2.0)
	Proposal with $(c_0, c_1) = (1/\sqrt{2}, 1/\sqrt{2})$		86.3 (4.4)	87.6 (3.4)	87.8 (3.2)
	Proposal with $(c_0, c_1) = (1, 0)$		87.4 (2.6)	87.7 (2.6)	87.9 (1.8)
	Proposal with $(c_0, c_1) = (0, 1)$		86.6 (2.5)	86.9 (2.2)	87.0 (1.3)

REFERENCES

1. Light RJ, Pillemer DB. *Summing up: The science of reviewing research.* Harvard University Press, 1984.
2. Duval S, Tweedie R. Trim and fill: a simple funnel-plot–based method of testing and adjusting for publication bias in meta-analysis. *Biometrics.* 2000;56(2):455–463.
3. Copas J. What works?: selectivity models and meta-analysis. *J R Stat Soc Ser A Stat Soc.* 1999;162(1):95–109.
4. Copas J, Shi JQ. Meta-analysis, funnel plots and sensitivity analysis. *Biostatistics.* 2000;1(3):247–262.
5. Copas J, Shi JQ. A sensitivity analysis for publication bias in systematic reviews. *Stat Methods Med Res.* 2001;10(4):251–265.
6. Heckman JJ. The common structure of statistical models of truncation, sample selection and limited dependent variables and a simple estimator for such models. In: , , National Bureau of Economic Research, 1976:475-492.
7. Heckman JJ. Sample selection bias as a specification error. *Econometrica.* 1979;47:153-161.
8. Copas JB. A likelihood-based sensitivity analysis for publication bias in meta-analysis. *J R Stat Soc Ser C Appl Stat.* 2013;62(1):47–66.
9. Begg CB, Mazumdar M. Operating characteristics of a rank correlation test for publication bias. *Biometrics.* 1994;50:1088–1101.
10. Egger M, Smith GD, Schneider M, Minder C. Bias in meta-analysis detected by a simple, graphical test. *BMJ.* 1997;315(7109):629–634.
11. Macaskill P, Walter SD, Irwig L. A comparison of methods to detect publication bias in meta-analysis. *Stat Med.* 2001;20(4):641–654.
12. Bürkner PC, Doebler P. Testing for publication bias in diagnostic meta-analysis: a simulation study. *Stat Med.* 2014;33(18):3061–3077.
13. Deeks JJ, Macaskill P, Irwig L. The performance of tests of publication bias and other sample size effects in systematic reviews of diagnostic test accuracy was assessed. *J Clin Epidemiol.* 2005;58(9):882–893.
14. Lin L, Chu H, Murad MH, et al. Empirical comparison of publication bias tests in meta-analysis. *Journal of general internal medicine.* 2018;33(8):1260–1267.
15. Hong C, Salanti G, Morton SC, et al. Testing small study effects in multivariate meta-analysis. *Biometrics.* 2020;76(4):1240–1250.
16. Luo C, Marks-Anglin A, Duan R, et al. Accounting for publication bias using a bivariate trim and fill meta-analysis procedure. *Statistics in medicine.* 2022;41(18):3466–3478.
17. Piao J, Liu Y, Chen Y, Ning J. Copas-like selection model to correct publication bias in systematic review of diagnostic test studies. *Stat Methods Med Res.* 2019;28(10-11):2912–2923.
18. Li M, Fan Y, Liu Y, Liu Y. Diagnostic test meta-analysis by empirical likelihood under a Copas-like selection model. *Metrika.* 2021;84:927–947.
19. Zhou Y, Huang A, Hattori S. A likelihood-based sensitivity analysis for publication bias on the summary receiver operating characteristic in meta-analysis of diagnostic test accuracy. *Stat Med.* 2023;42(6):781–798.
20. Zhou Y, Huang A, Hattori S. Nonparametric worst-case bounds for publication bias on the summary receiver operating characteristic curve. *Biometrics.* 2024;80(3):ujae080.
21. Reitsma JB, Glas AS, Rutjes AW, Scholten RJ, Bossuyt PM, Zwinderman AH. Bivariate analysis of sensitivity and specificity produces informative summary measures in diagnostic reviews. *J Clin Epidemiol.* 2005;58(10):982–990.
22. Hattori S, Zhou XH. Sensitivity analysis for publication bias in meta-analysis of diagnostic studies for a continuous biomarker. *Stat Med.* 2018;37(3):327–342.
23. Safdar N, Fine JP, Maki DG. Meta-analysis: methods for diagnosing intravascular device–related bloodstream infection. *Annals of Internal Medicine.* 2005;142(6):451–466.
24. Scheidler J, Hricak H, Kyle KY, Subak L, Segal MR. Radiological evaluation of lymph node metastases in patients with cervical cancer: a meta-analysis. *JAMA.* 1997;278(13):1096–1101.
